# Oxidative Stress and Erectile Dysfunction: Pathophysiology, Impacts, and Potential Treatments

**DOI:** 10.3390/cimb46080521

**Published:** 2024-08-14

**Authors:** Aris Kaltsas, Athanasios Zikopoulos, Fotios Dimitriadis, Danja Sheshi, Magdalena Politis, Efthalia Moustakli, Evangelos N. Symeonidis, Michael Chrisofos, Nikolaos Sofikitis, Athanasios Zachariou

**Affiliations:** 1 Third Department of Urology, Attikon University Hospital, School of Medicine, National and Kapodistrian University of Athens, 12462 Athens, Greece; ares-kaltsas@hotmail.com (A.K.); mxchris@yahoo.com (M.C.); 2 Department of Obstetrics and Gynecology, Royal Cornwall Hospital, Truro TR1 3LJ, UK; athanasios.zikopoulos1@nhs.net; 3 Department of Urology, Faculty of Medicine, School of Health Sciences, Aristotle University of Thessaloniki, 54124 Thessaloniki, Greece; helabio@yahoo.gr; 4 Department of Urology, Faculty of Medicine, School of Health Sciences, University of Ioannina, 45110 Ioannina, Greece; danjasheshi1@gmail.com (D.S.); politismagdalena@gmail.com (M.P.); nsofikit@uoi.gr (N.S.); 5 Laboratory of Medical Genetics, Faculty of Medicine, School of Health Sciences, University of Ioannina, 45110 Ioannina, Greece; thaleia.moustakli@gmail.com; 6 Department of Urology II, European Interbalkan Medical Center, 55535 Thessaloniki, Greece; evansimeonidis@gmail.com

**Keywords:** oxidative stress, erectile dysfunction, reactive oxygen species, nitric oxide signalling, endothelial dysfunction, antioxidants

## Abstract

Erectile dysfunction (ED) is a prevalent condition affecting men’s sexual health, with oxidative stress (OS) having recently been identified as a significant contributing causative factor. This narrative review aims to elucidate the role of OS in the pathophysiology of ED, focusing on impact, mechanisms, and potential therapeutic interventions. Key findings indicate that OS disrupts endothelial function and nitric oxide (NO) signaling, crucial for erectile function. Various sources of reactive oxygen species (ROS) and their detrimental effects on penile tissue are discussed, including aging, diabetes mellitus, hypertension, hyperlipidemia, smoking, obesity, alcohol consumption, psychological stress, hyperhomocysteinemia, chronic kidney disease, and sickle cell disease. Major sources of ROS, such as NADPH oxidase, xanthine oxidase, uncoupled endothelial NO synthase (eNOS), and mitochondrial electron transport, are identified. NO is scavenged by these ROS, leading to endothelial dysfunction characterized by reduced NO availability, impaired vasodilation, increased vascular tone, and inflammation. This ultimately results in ED due to decreased blood flow to penile tissue and the inability to achieve or maintain an erection. Furthermore, ROS impact the transmission of nitrergic neurotransmitters by causing the death of nitrergic neurons and reducing the signaling of neuronal NO synthase (nNOS), exacerbating ED. Therapeutic approaches targeting OS, including antioxidants and lifestyle modifications, show promise in ameliorating ED symptoms. The review underscores the need for further research to develop effective treatments, emphasizing the interplay between OS and vascular health in ED. Integrating pharmacological and non-pharmacological strategies could enhance clinical outcomes for ED patients, advocating for OS management in ED treatment protocols to improve patient quality of life.

## 1. Introduction

Erectile dysfunction (ED) is a common condition that affects millions of men globally, profoundly influencing their quality of life and overall well-being [1]. Numerous factors contribute to the pathophysiology of ED, including neurogenic, vascular, hormonal, and psychological elements, making it a complex condition [2]. Understanding these mechanisms provides insights into ED etiology and potential therapies [3].

The mechanisms underlying penile erection are intricate and involve a delicate balance between various physiological processes. Penile tumescence is primarily a neurovascular event regulated by the central and peripheral nervous systems. It heavily relies on the proper functioning of vascular and neural interactions. Nitric oxide (NO) plays a crucial role in these processes by facilitating smooth muscle relaxation in the corpora cavernosa, promoting vasodilation. This vasodilation is essential for the engorgement of the penis with blood [4].

The literature extensively covers the physiological mechanisms of penile erection and the impact of factors like endothelial dysfunction, reduced NO availability, and increased oxidative stress on ED. Key areas well represented in the literature include the roles of neurogenic, vasculogenic, hormonal, and psychological factors in ED pathophysiology, as well as the impact of lifestyle factors such as smoking, obesity, and alcohol consumption. Vasculogenic ED often stems from endothelial dysfunction, characterized by reduced NO availability and increased oxidative stress, which impede normal blood flow to penile tissue [5]. In contrast, neurogenic ED results from damage or dysfunction in the neural pathways involved in erection, commonly due to conditions such as diabetes, spinal cord injuries, or neurodegenerative diseases [6].

Oxidative stress, primarily due to the accumulation of reactive oxygen species (ROS), impairs endothelial function, reduces NO bioavailability, and promotes vascular dysfunction, which conditions all amount to key mechanisms in ED onset and progression. Recent studies have emphasized the significance of endogenous antioxidants in maintaining penile health and mitigating oxidative stress. Antioxidants, including enzymes like superoxide dismutase (SOD), catalase, and molecules such as glutathione, are vital in maintaining NO bioavailability. Their effect is primarily achieved through shielding NO from being scavenged by superoxide radicals [7].

Despite the existing body of literature, there are gaps and conflicting findings regarding the role of oxidative stress and the effectiveness of antioxidant therapies in ED [8]. This article aims to fill these gaps by providing a comprehensive review of the current literature on the role of oxidative stress in ED, its impact on endothelial and neural function, and potential therapeutic interventions targeting oxidative stress. Innovative therapeutic approaches, including high-dose antioxidants, hydrogen sulfide (H_2_S) and Angiotensin-(1–7), which have shown promise preclinical models, are discussed [9,10]. By synthesizing these findings, this review seeks to clarify the pathophysiological mechanisms of ED and propose novel directions for future research and treatment development, highlighting the groundbreaking nature of the literature search and findings.

## 2. Methods

### 2.1. Literature Search Strategy

The databases PubMed, Web of Science, Scopus, and Google Scholar were used to conduct a literature search encompassing the period from January 2000 to May 2024. The search was performed using specific keywords including “oxidative stress”, “erectile dysfunction”, “reactive oxygen species”, “nitric oxide signaling”, “endothelial dysfunction”, and “antioxidants”.

### 2.2. Inclusion and Exclusion Criteria

#### 2.2.1. Inclusion Criteria

- Peer-reviewed articles published between 2000 and 2024.
- Studies focusing on the pathophysiology of erectile dysfunction.
- Research discussing the impact of oxidative stress on erectile function.
- Articles addressing potential treatments for erectile dysfunction related to oxidative stress.
- Reviews, clinical trials, and meta-analyses related to oxidative stress and erectile dysfunction.

#### 2.2.2. Exclusion Criteria

- Non-English publications.
- Studies not focusing on the relationship between oxidative stress and erectile dysfunction.
- Articles lacking full-text access.
- Publications before 2000, unless they provide foundational knowledge.

### 2.3. Data Extraction and Synthesis

Three reviewers independently gathered data from each chosen study, including information on study design, sample size, demographic characteristics, major results, and conclusions. Any inconsistencies were addressed and resolved by reaching a consensus. A comprehensive synthesis was performed on the results of each study to provide a complete overview of the current understanding of oxidative stress and erectile dysfunction. This synthesis includes information on prevalence rates, causes, clinical symptoms, and prospective therapies.

### 2.4. Quality Assessment and Limitations

Each study included in the review was assessed for quality based on its design, sample size, and relevance to the review objectives. Potential limitations of this review include the presence of publication bias, scarcity of longitudinal data, and the use of small sample sizes in some studies. Future research should focus on conducting prospective studies with larger and more varied populations to better understand the progression and treatment of erectile dysfunction associated with oxidative stress.

## 3. Mechanisms of Penile Erection and Erectile Dysfunction

### 3.1. Physiology of Penile Erection

#### 3.1.1. Vascular and Neural Interactions

The erectile architecture of the penis consists of two chambers known as the corpora cavernosa. These chambers are made up of interconnected, densely packed sinusoids that span the length of the penis. They are encased by a semi-elastic membrane called the tunica albuginea [11]. Penile erection is a complex neurovascular process involving the central nervous system (CNS), peripheral nervous system, relaxation of corpora cavernosal smooth muscles, and vasodilation of blood vessels [12]. The maintenance of the erect or flaccid state is regulated by the relaxation or contraction of smooth muscle cells in the vicinity of the corpora cavernosa [13].

The CNS, in reaction to sensory inputs and cognitive stimuli, triggers the production of NO by neurons and endothelial cells surrounding the corpora cavernosa. Increased NO causes smooth muscle relaxation and dilation of blood vessels. This allows arterial blood flow into the penis to outpace the rate of venous outflow [14]. As a result, the obstructed blood accumulates, causing physical enlargement of sinusoidal spaces, increased pressure in the corpora cavernosa, and subsequently, tumescence of the penis [15]. The expanding sinusoid vessels are constricted by the tunica albuginea, and their continuous swelling compresses the walls of the draining venous structures. This further limits blood flow out of the penis, resulting in final firmness and maintaining the erection [16]. Detumescence occurs with reduced NO production, causing contraction of smooth muscle in the cavernosal region. This decreases arterial blood flow to the sinusoids, restoring equilibrium [17].

#### 3.1.2. The Crucial Role of Nitric Oxide (NO)

NO, a molecule crucial to erectile function, is produced by neuronal nitric oxide synthase (nNOS, NOS1), endothelial nitric oxide synthase (eNOS, NOS3), and immunoactivated macrophage-derived nitric oxide synthase (iNOS) [4,17]. Sensory reflexogenic and psychogenic sexual stimulation activate nNOS, within nitrergic nerve fibers derived from the major pelvic ganglia, which terminate in the penis [18]. The NO produced initiates the vasodilatory process. As blood volume increases, eNOS located in the endothelial layer of penile blood vessels and sinusoids is stimulated, leading to the continuous release of endothelial NO [18].

**Figure 1:**
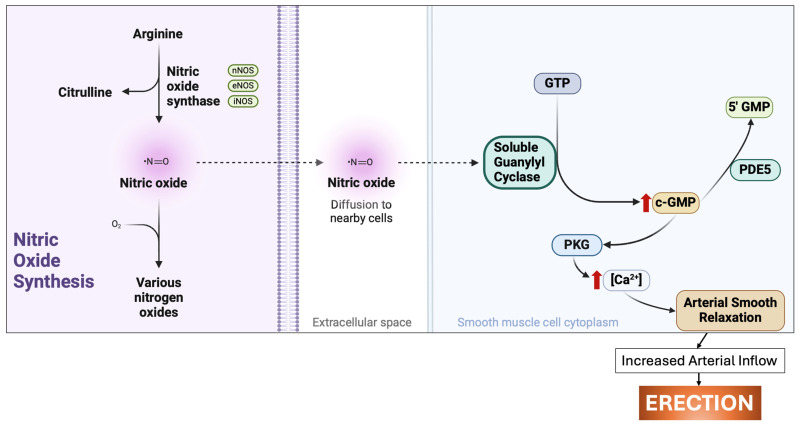
Nitric oxide signaling pathway in penile erection. NO: nitric oxide, nNOS: neuronal nitric oxide synthase, eNOS: endothelial nitric oxide synthase, iNOS: immunoactivated macrophage-derived nitric oxide synthase, c-GMP: cyclic guanosine monophosphate, 5′ GMP: 5′-guanosine monophosphate, GTP: guanosine triphosphate, PKG: protein kinase G, PDE5: phosphodiesterase type 5, Ca^2+^: calcium ions.

Following its synthesis, NO diffuses to adjacent smooth muscle cells in the penis and blood vessels. Here, it stimulates the activation of soluble guanylate cyclase (sGC), leading to the production of cyclic guanosine monophosphate (cGMP) [19]. The activation of cGMP triggers a cascade of protein kinase G-I (PKG-I)-dependent reactions. These reactions hyperpolarize and reduce calcium concentration in the cytosol of smooth muscle cells, making the cavernosal sinuses more compliant and inducing vasodilation [20]. As a result, blood flow to erectile tissue increases. The endothelium lining the sinusoid walls detects this stretch, further activating endothelial NOS in a positive feedback loop. This feedback is supported in part by the cyclic adenosine monophospate (cAMP) pathway in the endothelium lining the cavernous sinusoids and nearby penile arteries [4]. This process is illustrated in Figure 1, which shows the NO signaling pathway crucial for smooth muscle relaxation and vasodilation in penile erection.

The termination of penile erection is facilitated by the enzyme phosphodiesterase type 5 (PDE5) which breaks down cGMP into inactive 5′-GMP. PDE5 inhibitors, such as avanafil, sildenafil, tadalafil, and vardenafil, and blocks the action of PDE5, leading to an increase in cGMP levels and thereby predisposing the system towards penile erection (Figure 1) [13,21].

Moreover, NO regulates the generation of ROS and reactive nitrogen species (RNS) by means of S-nitrosylation, a mechanism that chemically alters proteins, safeguarding them from oxidative harm. This control aids in maintaining the redox equilibrium in penile tissues. For example, NO may hinder the nicotinamide adenine dinucleotide phosphate hydrogen (NADPH) oxidase complex, resulting in a decreased production of superoxide anions and a reduction in the synthesis of peroxynitrite, which can be harmful to cells [22]. In conditions such as sickle cell disease (SCD), the diminished availability of NO leads to reduced expression of PDE5, disrupting the regulation of penile erection and causing priapism. In such cases, treatments like haptoglobin therapy can increase NO availability and restore PDE5 expression and lower oxidative stress, thereby preventing priapism [22].

#### 3.1.3. Contractile Mechanisms in the Penis

The balance between contractile and relaxant factors in the penis determines the shift between penile erection and detumescence. Contractile factors include norepinephrine, endothelins, and angiotensins, while relaxant factors include NO, vasoactive intestinal peptide (VIP), and prostanoids. This balance governs the degree of cavernosal smooth muscle contraction and the status of the penis [23,24].

Penile smooth muscle contraction is regulated by mechanisms dependent on and independent of calcium channel activity [25]. In the flaccid state, endothelial signals (mainly prostaglandin and endothelin release) and direct sympathetic signaling (via norepinephrine release from cavernous nerves) change ion channel activity, increasing cytosolic calcium levels and causing tonic smooth muscle contraction [25]. Arousal induces NO release, cGMP activation, and the PK1 cascade, which hyperpolarize smooth muscle cells and reduce cytosolic calcium concentration. This reduction loosens the crossbridges between actin and myosin, relaxing smooth muscle and inducing vasodilation [26].

When cytosolic calcium levels return to baseline, the calcium-independent pathway becomes active. This pathway enhances calcium sensitivity without changing the amount of calcium in the cytosol. It involves the activation of RhoA, which binds to Rho-kinase (ROCK), leading to its activation. ROCK is crucial in regulating erectile function and maintaining penile flaccidity [27,28].

Studies have shown that prostanoid receptors, particularly thromboxane (TP) receptors, mediate the contraction of human trabecular smooth muscle and penile arteries, highlighting their role in regulating penile smooth muscle tone [29,30]. Additionally, the function of protein kinase C (PKC) is implicated in enhancing thromboxane receptor-mediated responses as well as impairing endothelially mediated relaxation in the corpora cavernosum, enhancing penile contractility [31].

### 3.2. Pathophysiology of Erectile Dysfunction

ED, as described by the Fourth International Consultation on Sexual Medicine, is the consistent or recurring inability to achieve and sustain an erection adequate for a fulfilling sexual experience [32]. ED is a common clinical problem worldwide, with an average prevalence ranging from 14% to 48% [33,34]. By 2025, the global prevalence of ED is estimated to exceed 300 million men [35]. ED pathophysiology is multifactorial, involving vascular, as well as hormonal, neurological, and psychological components [2,36]. The predominant molecular characteristics of ED are compromised production and function of NO, as well as heightened oxidative stress [37].

#### 3.2.1. Vascular-Related ED

ED often has a vascular component involving a decreased generation of vasorelaxant messengers, increased vasoconstriction, and reduced vasodilatory response in smooth muscle cells [38]. The primary contributing factor in vasculogenic ED is the decreased availability of NO in the endothelium, coupled with oxidative stress [7]. Systemic endothelial dysfunction can lead to reduced penile blood flow and subsequent erectile difficulties. Various risk factors, including diabetes, aging, high cholesterol levels, high blood pressure, high levels of homocysteine, lack of physical activity, sickle cell disease, smoking, atherosclerosis, hypertension, and metabolic syndrome, and other factors which contribute to endothelial dysfunction of vessels both inside and outside the penis, predispose individuals to the development of vasculogenic ED [39,40]. In vascular-related ED, increased oxidative stress results in reduced endothelial function due to decreased NO availability and heightened vasoconstriction.

Endothelial dysfunction is often an initial phase of vascular damage that may progress to more severe conditions like atherosclerosis in systemic vasculature. Clinically, this can manifest in coronary, renal, cerebral, and peripheral artery disorders. Vasculogenic ED is not only a covert indicator of cardiovascular and other systemic vascular illnesses but also poses an independent risk of future cardiovascular events. The association between vascular health and erectile function is well-established, with endothelial dysfunction being a local manifestation of systemic vascular issues [37].

#### 3.2.2. Neural-Related ED

Neurogenic ED arises from abnormalities in signal transfer between nerves that control the smooth muscle responses in the penis. This condition can result from nerve damage at any level of the nervous system, affecting both sensation and control. Conditions contributing to centralized neurogenic ED include Alzheimer’s disease, Parkinson’s disease, multiple sclerosis, stroke, and spinal cord injury. Meanwhile, peripheral causes may be primarily attributed to surgeries for prostate, bladder, and colon cancer. Long-term conditions such as diabetes can also damage neurons throughout the body, contributing to the development of neurogenic ED [41,42,43].

The molecular basis for neurogenic ED is not yet completely elucidated; however, several theories suggest potential causes. Key factors include impaired function of nNOS and reduced availability of neuronal NO. In neural-related ED, increased oxidative stress leads to dysfunctional neurotransmission and apoptosis of nitrergic nerves. Other contributing factors include the decrease in blood flow to nerve tissues and deficiencies in neurotrophic and growth factors [4,14].

## 4. Role of Oxidative Stress in Erectile Dysfunction

### 4.1. Oxidative Stress and ROS

#### 4.1.1. Definition and Impact of OS and ROS

Oxidative stress is a condition characterized by an imbalance between the production of ROS and the effectiveness of antioxidant systems in neutralizing these molecules [44]. ROS include free radicals like superoxide anions, hydroxyl, peroxyl, and hydroperoxyl radicals, as well as nonradical species like hydrogen peroxide and other peroxides [45]. Under normal physiological conditions, ROS are produced in a controlled manner and act as secondary messengers in various intracellular signaling pathways. However, excess ROS produced under pathophysiological conditions damage proteins, lipids, and DNA. This damage contributes to aging and various conditions, including cancers, vascular diseases, neurological disorders, and ED [46,47].

Antioxidants play a crucial role in mitigating the effects of oxidative stress and can be categorized into enzymatic and non-enzymatic types. Enzymatic antioxidants include SOD, catalase, glutathione peroxidase (GPx), and glutathione reductase (GR). Non-enzymatic antioxidants include vitamins A, C, and E, glutathione, uric acid, and polyphenols [48]. Enzymatic antioxidants are crucial in detoxifying ROS. Superoxide dismutase catalyzes the dismutation of superoxide into oxygen and hydrogen peroxide, which is then broken down by catalase and glutathione peroxidase [49]. Non-enzymatic antioxidants, such as glutathione and vitamins A, C, and E, play pivotal roles in neutralizing free radicals and protecting cellular components from oxidative damage [50]. This complex system of antioxidants ensures the neutralization of excess ROS, thereby maintaining cellular integrity and preventing oxidative damage.

#### 4.1.2. Cellular Sources of ROS

Primary sources of ROS production within cells are the enzymes NADPH oxidase, xanthine oxidase, the uncoupled form of eNOS, and the mitochondrial electron transport chain. These sources can interact, with the activity of one enhancing the function of the others [51,52,53].

##### NADPH Oxidase (Nox) Family

The NADPH oxidases are a family of transmembrane enzymes crucial for generating ROS within biological systems. These enzymes transfer electrons to molecular oxygen from cytosolic NADPH, producing superoxide anions [54]. There are seven distinct NADPH oxidase isoforms in mammalian cells. The prototypical NADPH oxidase contains cytosolic components (p47phox, p67phox, and similar proteins) and membrane-bound components (p22phox and gp91phox). Upon stimulation, these subunits come together to form the activated enzyme complex [55,56].

NADPH oxidases can be activated by various stimuli, including angiotensin II, proinflammatory cytokines, vasoconstrictors, growth factors, hypoxia, mechanical cell stimuli, and metabolic factors like hyperglycemia, advanced glycation end products (AGEs), elevated free fatty acid levels, hyperinsulinemia, and even existing superoxide levels. ROS generated by NADPH oxidases are essential for certain host defense mechanisms, such as neutrophil function in eradicating pathogens [57]. However, excessive ROS production by NADPH oxidases can cause oxidative stress, damaging healthy tissues and contributing to inflammatory diseases like rheumatoid arthritis and inflammatory bowel diseases [58]. Additionally, these ROS are linked to aging and several disorders, including hypertension, diabetes mellitus, hypercholesterolemia, and SCD. Recent data indicate that NADPH oxidase contributes to the pathophysiology of various ED conditions [59].

##### Xanthine Oxidase

Xanthine oxidase (XO) is an enzyme responsible for catalyzing the conversion of hypoxanthine to xanthine, and subsequently to uric acid. During this process, it generates ROS, including hydrogen peroxide and various superoxides [60,61]. This multifaceted enzyme is involved in various physiological processes, including the generation of ROS and the production of uric acid. While the molecular origin of XO is not fully understood, elevated serum cholesterol, liver injury, general inflammation, and hypoxic conditions may trigger heightened enzyme secretion by hepatic and other visceral mechanisms into the bloodstream [62].

Once in general circulation, XO attaches to the surface of endothelial cells and triggers superoxide generation. Endothelial cells, however, also produce endogenous XO [63]. Increased expression and activity of XO have been linked to endothelial dysfunction in animal model studies of SCD, hypertension, diabetes mellitus, and hypercholesterolemia [64]. Additionally, xanthine oxidase is linked to the regulation of nuclear factor of activated T-cells 5 (NFAT5) target genes through ROS, regulating gene expression in different cellular contexts [65].

The enzyme’s role in generating ROS within the vasculature has been found to be integral to the endothelial component of the inflammatory process [66]. However, XO involvement in cardiovascular disorders in humans remains a topic of debate. Similarly, the precise role of ROS formed by xanthine oxidase in the penis is not well understood, and investigation is necessary to ascertain whether it contributes to ED.

##### eNOS Uncoupling

Physiologically, NOS isoforms generate NO, which is vital for vasodilation, antioxidation, and the maintenance of vascular homeostasis. However, in pathological situations, NOS isoforms may transform into prooxidants, predisposing the body toward superoxide production rather than NO. This process, referred to as NOS uncoupling, describes a transition in the activity of several NOS isoforms, including constitutive eNOS, nNOS, and iNOS. NOS uncoupling results in reduced synthesis of NO and enhances the capability of the enzyme to produce ROS [67].

eNOS uncoupling is particularly significant in the context of cardiovascular diseases, where it leads to superoxide anion production instead of NO [68]. Several mechanisms contribute to the uncoupling of eNOS, including deficiency of the cofactor tetrahydrobiopterin (BH4), depletion of the substrate L-arginine, and accumulation of asymmetric dimethylarginine (ADMA), a competitive inhibitor of eNOS [69]. Additionally, the lack of BH4, S-glutathionylation, as well as alterations in the eNOS dimer–monomer ratio have been identified as key factors leading to eNOS uncoupling [70,71,72].

Oxidative stress, hypoxia, and reoxygenation all trigger xanthine oxidase-mediated superoxide generation. This results in tetrahydrobiopterin depletion and S-glutathionylation, ultimately leading to eNOS uncoupling [73]. Sub-optimal action of the complex regulatory mechanisms governing the interplay between BH4 oxidation and glutathionylation of eNOS cysteine residues may also induce eNOS uncoupling, highlighting the complex regulatory mechanisms involved in this process [74]. This dysregulation of eNOS uncoupling has been implicated in various pathological conditions, including atherosclerosis, diabetes mellitus, hypertension, ischemia–reperfusion injury, and heart failure [75].

Disrupted eNOS function is linked to endothelial dysfunction, highlighting the significant impact eNOS uncoupling has on vascular health [76]. Specifically, eNOS uncoupling has been linked to increased oxidative stress, inflammation, and vascular pathogenesis, amounting to a key contributor to cardiovascular diseases [77]. Recent research has shown that the uncoupling of eNOS in the penis also plays a major role in causing ED and regional oxidative stress [78].

##### Mitochondrial Electron Transport

Mitochondria produce ROS as a byproduct of regular oxidative phosphorylation and adenosine triphosphate (ATP) generation due to electron leakage [79]. This electron transport chain (ETC), located in the inner mitochondrial membrane, involves the sequential transfer of electrons through complexes I to IV, with molecules like ubiquinone mediating electron transport and ATP synthesis [80]. The ETC is essential for generating the majority of cellular ATP through oxidative phosphorylation. However, disruptions in electron flow can lead to severe mitochondrial dysfunction and disease states [81]. Additionally, the ETC regulates cellular oxygen availability and impacts the stabilization of proteins like hypoxia-inducible factor-1α under hypoxic conditions [82,83].

Giacco and Brownlee conducted studies indicating that hyperglycemia-induced oxidative stress originating from mitochondria is the first step in the progression of diabetic vascular problems [84]. However, due to the toxicity of mitochondrial inhibitors and the absence of experimental animal models, our understanding of the contribution of excessive mitochondrial ROS to in vivo disease is limited. Dysfunctional mitochondria may produce insufficient ATP, high levels of ROS and proapoptotic factors, contributing to the pathogenesis of various disorders, including neurodegenerative diseases [85].

The ETC is susceptible to damage during conditions such as ischemia and reperfusion, where interruptions in mitochondrial respiration can exacerbate cardiac injury. Pharmacologic inhibition of electron transport during early reperfusion reduces myocardial injury, indicating the potential usefulness of modulating electron transport to safeguard cardiac mitochondria [86]. The involvement of superoxide produced by mitochondria in erectile dysfunction has not been extensively studied, suggesting a further avenue of research for the pathophysiologic and therapeutic evaluation on ED.

### 4.2. Impact of Oxidative Stress on ED

Oxidative stress is linked to both vasculogenic as well as neurogenic ED, with the former being more extensively studied. Superoxides produced by vascular sources within the penis react with NO to create RNS like peroxynitrite, which causes oxidative DNA damage and disrupts lipids and proteins [46]. This contributes to endothelial dysfunction by impairing NO availability.

ROS produced in smooth muscle and endothelial cells can scavenge NO, influencing the expression and activity of eNOS [47]. ROS also deplete cofactors necessary for NOS function, generate vasoconstrictors, reduce smooth muscle cell integrity, deactivate antioxidants, and cause structural and functional alterations in blood vessels [11]. Elevated oxidative stress can contribute to atherosclerosis by oxidizing low-density lipoproteins (LDL), the primary cholesterol transporter in the blood, and increasing superoxide production [87].

**Figure 2:**
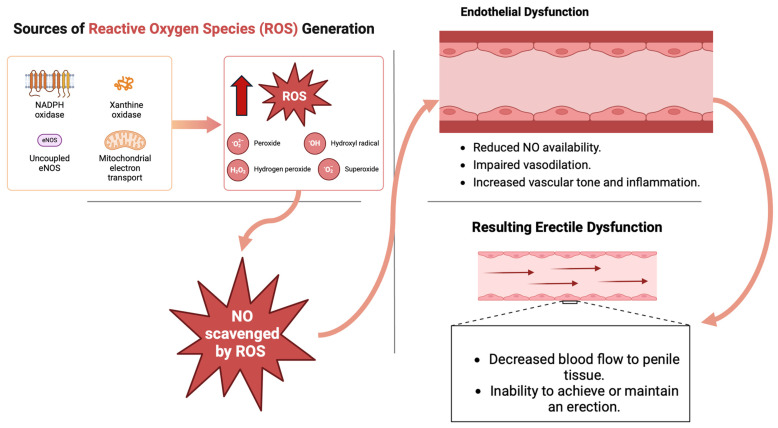
Impact of oxidative stress on erectile dysfunction. ROS: reactive oxygen species, NO: nitric oxide.

ROS impact the transmission of nitrergic neurotransmitters by causing the death of nitrergic neurons and reducing the signaling of nNOS, leading to ED [6]. Figure 2 illustrates the sources of ROS, including NADPH oxidase, xanthine oxidase, uncoupled eNOS, and mitochondrial electron transport. NO is scavenged by these ROS, leading to endothelial dysfunction characterized by reduced NO availability, impaired vasodilation, increased vascular tone, and inflammation. This ultimately results in erectile dysfunction due to decreased blood flow to penile tissue and the inability to achieve or maintain an erection.

Outlined in the following sections are the established origins of ROS and the specific areas in the penis where ROS exert their effects on vasculogenic and neurogenic ED related to age and other medical conditions [88].

### 4.3. Influencing Factors and Their Molecular Mechanisms

#### 4.3.1. Aging

Aging significantly contributes to age-associated ED through declining androgen levels and increased OS, leading to endothelial dysfunction, smooth muscle cell apoptosis, and inhibited NO production, all crucial factors in ED development [89]. Research shows that endothelial and smooth muscle cells in the penis of elderly rats generate ROS [90,91,92,93]. Specific mechanisms stimulating ROS production in the aging penis remain largely unknown, but evidence suggests that eNOS uncoupling plays a significant role. Sepiapterin, a precursor in BH4 biosynthesis, can inhibit age-related ED and decrease OS by preventing eNOS uncoupling [90]. Further research is needed to understand the exact mechanisms of eNOS uncoupling and other ROS sources in age-related ED.

Disruptions in central and peripheral neurotransmission also contribute to reduced corpora cavernosa relaxation with age. Central neuropathy involves excessive ROS generation and apoptosis in hypothalamic regions regulating penile erection, while peripheral processes include degeneration of penile nitrergic nerve fibers and inhibition of nNOS [94]. However, the exact origins of ROS and the mechanisms by which they damage nitrergic neurotransmission in age-related ED remain unidentified.

#### 4.3.2. Chronic Health Conditions

##### Diabetes Mellitus

Oxidative stress is a significant factor in diabetes mellitus-associated ED. Studies show a high prevalence of ED in individuals with diabetes, with hyperglycemia and hyperlipidemia enhancing ROS formation [95]. Penile tissue and blood samples from males with diabetes-associated ED [96,97,98,99] and animals with type 1 diabetes (T1D) show elevated superoxide levels [100,101,102,103,104,105,106,107]. OS hampers NO production by neurons and endothelial cells in the penis, leading to increased apoptosis and fibrosis of cavernosal tissue [31,108,109,110]. It also causes nerve injury through membrane lipid peroxidation [111]. DNA damage triggers signaling molecules, resulting in increased production of proinflammatory chemicals and suppression of eNOS activity [112,113]. Elevated expression of the NADPH oxidase component p47phox in rats with T1D suggests its role in generating ROS [114].

Research on oxidative stress in type 2 diabetes (T2D) animal models is limited [115], but studies reveal that rats and mice with T2D have reduced antioxidant levels in their penises, suggesting increased oxidative stress [116]. Diabetes is also linked to gradual degeneration of penile nitrergic neurons. In rats with T1D, an initial reversible drop in nNOS levels is observed in nitrergic penile nerves. As diabetes progresses, advancing apoptosis of the nitrergic nerves in the major pelvic ganglion (MPG) generally follows, attributed to severe oxidative stress [117,118]. Various studies have explored using N-acetylcysteine, an oxidative stress inhibitor, to improve diabetes-associated ED by inhibiting oxidative stress [46].

##### Hypertension

Research has revealed a high prevalence of ED in individuals with hypertension, with around 30% of male patients being affected [119]. Angiotensin II is a powerful vasoconstrictor involved in the development and maintenance of high blood pressure. When Angiotensin II binds to the angiotensin I (AT1) receptor in the arterial wall, it triggers the activation of NADPH oxidase, leading to the formation of ROS [120]. Hypertensive rats have elevated levels of lipid peroxidation in the corpora cavernosa [121,122,123]. The protein levels of NADPH oxidase subunits p47phox [124] and gp91phox [123] are elevated in the penis of hypertensive rats, alongside increased oxidative stress and ED. Additionally, apocynin, a NADPH oxidase inhibitor, has been found to reduce oxidative stress while also enhancing normal erectile function in hypertensive rat models [124]. ROS and NADPH oxidase contribute to the pathophysiology of ED, suggesting that oxidative stress is directly causal in hypertension-associated ED [125].

##### Hyperlipidemia

Hyperlipidemia significantly increases the risk of developing vasculogenic and neurogenic ED. Oxidative stress is a major contributing factor to the development of vasculogenic ED associated with hyperlipidemia [126]. Tissue from the corpora cavernosa of animals on a high-cholesterol diet showcases increased ROS production [127,128]. In the penile tissue of mice, hypercholesterolemia leads to increased expression of the NADPH oxidase subunits p47phox, p67phox, and gp91phox [129]. Meanwhile, in the same models, NADPH oxidase inhibition using compounds such as diphenyleneiodonium chloride and apocynin reduces the production of ROS and has a protective effect on erectile function [129]. In penile tissues of animals with hypercholesterolemia, experiments indicate that eNOS uncoupling [129,130] acts as a further ROS source but xanthine oxidase does not [131]. LDL oxidation is a major factor in the development of ED in hyperlipidemia, with high levels of oxidatively modified LDL (oxLDL) observed in penile tissue from patients [132] and animal models [130]. In systematic vasculature, oxLDL increases the production of superoxides by inducing the uncoupled form of eNOS, the enzymes NADPH oxidase and xanthine oxidase, and the mitochondrial electron transport chain; however, its local function in penile vasculature has yet to be elucidated [133]. Experimental studies have indicated therapeutic potential in the compound sodium tanshinone IIA sulfonate for attenuating ED in rat models of hyperlipidemia [134]. This compound improves endothelial function, reduces oxidative stress, and modulates lipid metabolism, contributing to its efficacy in managing ED in the context of hyperlipidemia.

##### Chronic Kidney Disease (CKD)

CKD is significantly linked to ED, affecting up to 70% of men with CKD, especially those on dialysis or who have had a kidney transplant [135]. CKD causes endothelial dysfunction by reducing NO production and increasing oxidative stress, which impairs vasodilation and blood flow to penile tissue [136]. Accumulation of uremic toxins in CKD can lead to peripheral and autonomic neuropathy, affecting nerve signals required for erections [137]. CKD-related disruptions in the hypothalamic–pituitary–gonadal axis result in decreased testosterone levels, reducing sexual desire and erectile capability [138]. Impaired renal clearance may also elevate prolactin levels, further hindering gonadotropin-releasing hormone (GnRH) function and exacerbating hypogonadism [139]. Furthermore, the chronic nature of CKD, along with its symptoms and activity restrictions, can cause psychological stress, depression, and anxiety, which all contribute to ED in and of themselves. The interconnectedness of these factors clearly underscores the complexity of ED management in CKD patients [140].

#### 4.3.3. Lifestyle and Behavioral Factors

##### Smoking

Smoking cigarettes releases substantial quantities of chemical components, including NO, ROS, peroxynitrite, and free radicals derived from organic molecules. On entering systemic circulation, these components can subsequently stimulate the generation of ROS in blood vessels [141]. More specifically, within the circulatory system, cigarette smoking increases the production of superoxide mediated by uncoupled eNOS and NADPH oxidase in smooth muscle as well as endothelial cells. Additionally, the release of proinflammatory cytokines increases, while also stimulating RhoA/ROCK pathways that lead to muscular contraction. Consequently, there is a decrease in the availability of nitric oxide, increase in blood vessel constriction, and overall impairment of endothelial function. Both active and passive cigarette smoking are considered risk factors for ED [142,143].

Emerging scientific research indicates that smoking-related ED is linked to a decrease in the availability of NO caused by heightened oxidative stress. Long-term exposure to cigarette smoke hampers the brain’s capacity to regulate penile erection and reduces the availability of NO [144]. In cigarettes, nicotine has been identified as the component primarily responsible for the vascular consequences resulting from persistent smoking [145]. Nicotine induces NADPH oxidase, increasing superoxide production in rabbit cavernosal smooth muscle cells [146].

##### Obesity

Obesity is a major risk factor for ED, involving multiple physiological processes including oxidative stress, inflammation, and endothelial dysfunction [147]. Obesity leads to the release of pro-inflammatory substances like tumor necrosis factor alpha (TNF-α), interleukin-6 (IL-6), and C-reactive protein (CRP), which induce the generation of ROS, resulting in elevated oxidative stress [148]. Increased ROS is associated with decreased NO levels, and impaired endothelial cell function. This endothelial dysfunction, characterized specifically by reduced NO production, decreases blood vessel dilation and blood flow to penile tissue, contributing to ED [149].

Obesity also alters hormone levels by converting androgens into estrogens, leading to decreased testosterone. Low testosterone (hypogonadism) is common in obese males and can diminish sexual desire and impair erectile function [150]. Additionally, obesity increases the risk of insulin resistance and T2DM, both of which predispose individuals toward ED by exacerbating oxidative stress and endothelial dysfunction [88].

##### Alcohol Consumption

Excessive alcohol use is a significant contributor to ED through oxidative stress, endothelial dysfunction, hormonal imbalances, and neurovascular changes [151]. Chronic alcohol use stimulates ROS generation, leading to oxidative stress that harms endothelial cells and overall vasoconstriction, resulting in restricted blood flow to the penis [152]. Furthermore, cytochrome P450 2E1 (CYP2E1) plays a crucial role in alcohol metabolism and increases ROS production, contributing to endothelial dysfunction [153]. Prolonged alcohol use is also linked to hormonal imbalances, including lower testosterone and higher estrogen levels, resulting in hypogonadism and reduced sexual drive [154].

Additionally, oxidative stress from alcohol use may harm the nervous system, impairing the neural mechanisms responsible for erection by damaging neurons involved in NO release [155]. Multiple studies have demonstrated a clear correlation between alcohol intake and ED. Most notably, a meta-analysis revealed a J-shaped correlation, indicating that moderate alcohol use might be protective, but excessive consumption significantly increases ED risk [156]. This effect may be substantiated by another study, which, having found a high prevalence of ED in men with alcohol use disorder (AUD), identified significant improvement after a month of abstinence [157].

##### Psychological Stress

Psychological stress activates the hypothalamic–pituitary–adrenal (HPA) axis, resulting in elevated cortisol levels, disrupting the balance of sex hormones essential for libido and erectile function [158]. It also activates the sympathetic nervous system, releasing catecholamines, chiefly adrenaline, which causes vasoconstriction, reducing penile blood flow and complicating erection maintenance [159].

Chronic psychological stress is linked to increased production of ROS and inflammatory cytokines, leading to endothelial dysfunction in time. This endothelial dysfunction, in turn, impairs the release of NO, which is essential to the appropriate perfusion of penile tissue with blood [160,161]. Furthermore, stress-related anxiety and depression negatively impact mood, motivation, and overall mental well-being, leading to decreased libido and performance anxiety, exacerbating ED [162].

#### 4.3.4. Genetic Disorders

##### Hyperhomocysteinemia

ED is associated with elevated homocysteine levels in both human and animal studies [163]. It has been observed that in the rabbit corpora cavernosa, hyperhomocysteinemia impairs relaxation by reducing the availability of endothelial NO and increasing the production of superoxide [164,165]. Nevertheless, the specific processes by which hyperhomocysteinemia triggers ROS generation and the specific ROS origins remain unidentified.

##### Sickle Cell Disease

SCD is characterized by the production of aberrant sickle hemoglobin (HbS), causing red blood cells to become stiff, impairing blood circulation and reducing penile blood supply [166]. This dysfunction is characterized by anomalies in the availability of NO, heightened reactions to vasoconstrictors, and increased oxidative stress [167,168,169]. Priapism, a prevalent vascular disorder associated with SCD, affects around 40% of male SCD patients [170,171]. Research in mice with SCD and rats with priapism found increased protein oxidation and lipid peroxidation in the corporal tissue [172].

To provide a comprehensive overview, Table 1 below summarizes the primary sources of ROS and their mechanisms, associated conditions, and relevant clinical and experimental models in the context of ED.

## 5. Antioxidants and Therapeutic Strategies for Erectile Dysfunction

### 5.1. Endogenous Antioxidants in Penile Health

Endogenous antioxidants are essential for maintaining penile health and function by reducing oxidative stress, a significant factor in the development of ED. Superoxide dismutase (SOD) is crucial for dismutating superoxide radicals into hydrogen peroxide, thereby protecting NO from being scavenged. Catalase then converts hydrogen peroxide into water and oxygen, further reducing oxidative stress. Glutathione (GSH) acts as a major antioxidant by directly neutralizing reactive oxygen species and regenerating other antioxidants, which are crucial for preserving the bioavailability and functionality of NO by protecting it from being scavenged by superoxide radicals [173].

Rat penile endothelial and smooth muscle cells have a reservoir of antioxidants, including reduced glutathione, catalase, glutathione peroxidase, and SOD3 [174,175,176]. Cavernosal blood and tissue also contain antioxidants such as glutathione, vitamin C, bilirubin, albumin, and uric acid [177]. However, studies have yielded conflicting findings regarding levels of natural antioxidants in affected penile tissue. In some animal models of ED, the concentration of reduced glutathione drops, while cytoplasmic SOD expression increases. In contrast, extracellular levels of SOD protein, SOD activity, and RNA expression remain constant [91,92].

In individuals with diabetes, penile tissue contains reduced levels of catalase mRNA, while levels of glutathione vary, being either lowered or elevated [99,107,178]. Meanwhile, in diabetic rat penises, mRNA expression of SOD2 is reduced, though the activity of SOD remains constant [178]. Hypertensive rats further demonstrate reduced SOD activity in the corpora cavernosa [121,122,123], whereas animals on a high-cholesterol diet exhibit enhanced SOD activity as well as unaltered glutathione peroxidase and catalase activity [179]. Up to this point, however, there is a lack of reliable and clear evidence on the penile antioxidant status as related to ED.

### 5.2. Therapeutic Strategies to Mitigate Penile Oxidative Stress

#### 5.2.1. Role of Antioxidants in Penile Oxidative Stress Reduction

Antioxidants effectively counteract oxidative stress, protecting penile function [88,180]. High doses of vitamins C and E may reverse some of the adverse effects associated with smoking, particularly regarding NO levels [181,182]. Antioxidants also help regress Peyronie’s disease plaques and reduce penile fibrosis [183]. Long-term treatment with Angiotensin-(1–7) has been found to reduce penile fibrosis by attenuating oxidative stress [184]. Hydrogen sulfide (H_2_S) supports penile function by scavenging ROS or stimulating antioxidant defenses [185].

The impact of antioxidants on ED has been extensively assessed in various animal models. Introducing SOD by gene transfer in elderly mice decreased the production of superoxide anions and restored normal erectile function [92,186]. Antioxidants such as SOD, ascorbic acid, vitamin E, melatonin, alpha-lipoic acid, peroxynitrite decomposition catalyst, and gamma-linolenic acid have been identified as attenuating autonomic neuropathy and diabetic vasculopathy in the penis, thereby improving overall erectile function to various extents [186,187,188,189,190,191]. SOD and catalase have been further found to mitigate superoxide generation in the penis caused by hyperhomocysteinemia [164,165].

However, a further fundamental scientific and clinical investigation is necessary to elucidate the size of the effect of antioxidant treatments for oxidative stress affecting the penis, with the aim of establishing a solid scientific foundation for potential therapeutic use [186]. Currently, antioxidant treatments are not considered suitable to treat ED. There is growing awareness within the scientific community that interventions which scavenge ROS in a non-specific manner do not necessarily reverse disease effectively. This has been demonstrated in the case of ED and various cardiovascular pathologies [47].

Multiple randomized clinical studies have shown that long-term use of antioxidants, such as vitamins E and C, does not consistently prevent cardiovascular events and may even elevate the risk of heart failure in individuals with pre-existing vascular diseases [192]. This ineffectiveness may be due to the antioxidants being insufficiently available in the right place at the right time, or due to their unintended disruption of physiological functions controlled by ROS [193].

In conclusion, controlled and targeted ROS scavenging by antioxidant interventions represents a critical avenue for therapeutic strategies aimed at mitigating penile oxidative stress. Reducing oxidative stress potentially preserves penile function, prevents fibrosis, and improves overall penile health. However, further research is needed to optimize the delivery and efficacy of antioxidant treatments for ED and other oxidative stress-related penile conditions.

#### 5.2.2. Targeting NADPH Oxidase in Penile Tissue

##### PDE5 Inhibitors and Their Mechanisms

PDE5 inhibitors, such as sildenafil citrate, tadalafil, avanafil, and vardenafil hydrochloride, are widely recognized for their effectiveness in treating ED by enhancing penile erection during sexual stimulation [194,195]. They accomplish this by inhibiting the breakdown of cGMP catalyzed by PDE5, thereby facilitating smooth muscle relaxation, enhancing blood flow to the penis, and sustaining erection [196].

Recent studies have expanded the understanding of PDE5 inhibitor effects, revealing their additional benefits in reducing ROS generation and inhibiting ROS-mediated upregulation of penile PDE5 [197]. Multiple animal studies indicate that PDE5 inhibitors can significantly reduce oxidative stress in penile tissues. For example, sildenafil citrate has been shown to decrease the formation of superoxide in mouse models of ED caused by secondhand smoking exposure [198]. Similarly, sildenafil reduces oxidative stress in the smooth muscle of rabbit penile blood vessels exposed to substances which induce ROS production such as TNF-α, nicotine, homocysteine–copper combination, and the thromboxane A2 mimic [146,199,200]. This effect is believed to result from sildenafil’s ability to inhibit NADPH oxidase by reducing the protein production of its component p47phox [200].

Furthermore, PDE5 inhibitors prevent the PDE5 upregulation following oxidative stress in the penis, which would otherwise lead to ED via depletion of cGMP levels. The development as well as the activity of PDE5 in penile blood vessels is enhanced by oxidative stress [201]. In studies with animal models experiencing increased oxidative stress, the use of sildenafil prevented PDE5 overexpression via NADPH oxidase inhibition. Therefore, the therapeutic advantage of PDE5 inhibitors is partly achieved by inhibiting PDE5 overexpression as well as reducing NADPH oxidase-derived oxidative stress, both mechanisms impairing normal erection [146,199,202].

As regards other sildenafil derivatives, in rabbits with hypercholesterolemia, the derivative sildenafil nitrate (NCX 911), which acts as a NO donor, has been shown to suppress superoxide generation via NADPH oxidase more effectively than sildenafil citrate [131]. This enhanced effect is attributed to the antioxidant properties of externally introduced NO [203]. Additionally, ACS6, another sildenafil derivative which acts as a hydrogen sulfide donor, prevents the production of superoxides in the erectile tissues of rabbits with hypercholesterolemia, blocking the subunit p47phox of the enzyme NADPH oxidase as well as activating pathways, including PKA and PKG [202]. Tadalafil also positively impacts the cardiovascular system by decreasing oxidative stress levels in individuals with ED [204,205]. Studies suggest that combining PDE5 inhibitors with vasorelaxant drugs effectively reduces oxidative stress and enhances the erection compared to PDE5 inhibitors as monotherapy [202,206].

In summary, PDE5 inhibitors not only enhance NO signaling and cGMP levels but also significantly contribute to the mitigation of penile oxidative stress [207]. This dual action makes them an essential therapeutic step for improving erectile function by addressing both the biochemical pathways and oxidative damage underlying ED.

##### Angiotensin-Converting Enzyme Inhibitors and AT1 Receptor Blockers

Angiotensin II contributes to endothelial dysfunction, causing oxidative stress by NADPH oxidase activation (via the AT1-receptor). AT1-receptor blockers and ACE-inhibitors reduce oxidative stress by inhibiting NADPH oxidase activity and enhancing ROS removal [208]. Several clinical studies have indicated that inhibiting angiotensin II signaling positively impacts endothelial function in hypertensive individuals and those with metabolic syndrome, leading to decreased mortality, myocardial infarction, and stroke [209,210].

Numerous studies have demonstrated that anti-hypertensive treatments using ACE inhibitors and AT1 receptor blockers positively impact erectile function, both in male individuals and animal models with ED [211]. AT1-receptor blockers like losartan and irbesartan reduce ROS formation and enhance NO production in penile tissues of elderly rats and mice with hypercholesterolemia, independently of their impact on blood pressure [212,213]. This suggests that improved erectile function might be directly attributed to suppressed ROS formation.

These agents also influence the activation of Cu/Zn-containing superoxide dismutase, an enzyme involved in scavenging ROS in hypertensive conditions [214]. By affecting the activity of ROS-scavenging enzymes, ACE-inhibitors and AT1-receptor blockers reduce oxidative stress within penile tissues, potentially improving erectile function and overall penile health. Additionally, these agents benefit the cardiovascular and renal systems, highlighting their role in mitigating oxidative damage and improving organ function [215,216]. They have shown promise in regressing left ventricular hypertrophy and preventing aging-related endothelial dysfunction, supporting their potential in combating oxidative stress-related complications.

The ability of ACE-inhibitors and AT1-receptor blockers to modulate the renin–angiotensin system and affect various cellular responses, including ROS production, suggests a broader impact on oxidative stress beyond the cardiovascular system. By targeting specific points in the renin–angiotensin system, these agents reduce the detrimental effects of oxidative stress on penile tissues.

In conclusion, ACE-inhibitors and AT1-receptor blockers present valuable potential therapeutic interventions for mitigating penile oxidative stress. By elucidating the mechanisms whereby these agents influence organ function through oxidative stress, the approach contributes to a more comprehensive understanding of the role of pharmacological interventions in preserving penile health, and improving erectile function can be achieved.

##### Statins and Their Effects on Penile Oxidative Stress

Statins evince beneficial effects beyond their cholesterol-reducing properties by improving endothelial function through reducing oxidative stress and increased eNOS activity. Statins achieve these benefits by upregulating antioxidants such as SOD3 and catalase by preventing superoxide generation in endothelial cells [217,218,219]. This is accomplished by suppressing NADPH oxidase and preventing eNOS uncoupling [219,220].

Research has demonstrated that certain statins, including rosuvastatin and atorvastatin, can improve ED associated with diabetes, metabolic syndrome, and hypertension [221]. These statins inhibit RhoA/ROCK signaling in penile tissue, thereby increasing the efficacy of sildenafil. However, clinical studies examining the effects of statins in men with ED yield inconsistent results. Some studies indicate that atorvastatin can enhance erectile performance in men, both alone and in conjunction with sildenafil [222,223,224], while other studies do not support this finding [225,226].

The precise mechanisms underlying the positive effects of statins on erectile function, specifically, whether they are due to cholesterol reduction or other mechanisms, remain unclear. In addition, the potential reduction in oxidative stress in the penis due to statin therapy has yet to be extensively studied. More fundamental and clinical research is needed in determining whether statins enhance erectile performance through mitigation of oxidative stress and improved penile endothelial function. Furthermore, specifics of the mechanisms behind these potential benefits require further investigation.

**Figure 3:**
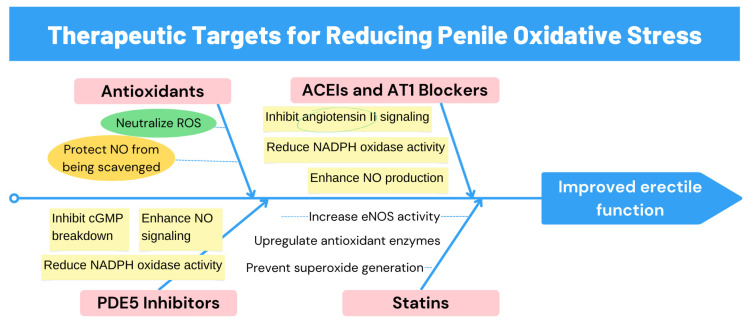
Therapeutic targets for reducing penile oxidative stress. ROS: reactive oxygen species, NO: nitric oxide, PDE5: phosphodiesterase type 5, ACE: angiotensin-converting enzyme, AT1: angiotensin II type 1 receptor.

Figure 3 identifies therapeutic targets to reduce oxidative stress in penile tissue, highlighting therapeutic strategies to improve erectile function. Antioxidants neutralize ROS, protecting NO from being scavenged. PDE5 inhibitors enhance NO signaling and reduce NADPH oxidase activity. ACE inhibitors and AT1 blockers inhibit angiotensin II signaling and enhance NO production. Statins increase eNOS activity, upregulate antioxidant enzymes, and prevent superoxide generation.

#### 5.2.3. Targeting eNOS Uncoupling to Improve Penile Function

Tetrahydrobiopterin (BH4), a crucial cofactor for NOS, and its precursor sepiapterin, are frequently used as inhibitors of oxidative stress and eNOS uncoupling [227]. They also contribute to the prevention of cardiovascular diseases [227]. Several extensive clinical studies are currently assessing the effectiveness of oral BH4 in treating systemic hypertension, peripheral arterial disease, coronary artery disease, pulmonary arterial hypertension, and SCD. Additionally, researchers are testing numerous medications on their potential to enhance BH4 bioavailability, which is considered a superior treatment approach for eNOS coupling compared to direct BH4 supplementation [228].

Current therapeutic agents in cardiovascular medicine, including statins, erythropoietin, folic acid, insulin, ascorbic acid, and angiotensin II signaling inhibitors, restore eNOS function by promoting BH4 binding to NOS, increasing BH4 production, or protecting BH4 from oxidation [229]. However, data supporting improvement in erectile function through specifically targeting eNOS uncoupling are insufficient.

Administering folic acid to diabetic rabbits has been demonstrated to reduce oxidative stress within cavernosal tissue [103]. Recent research indicates that supplementation with sepiapterin can prevent penile oxidative stress and maintain normal penile erection in older rat models [90]. Further research is necessary to investigate the potential therapeutic benefits of regulating eNOS uncoupling on penile erection in individuals with medical conditions and ED.

#### 5.2.4. Natural Antioxidant Beverages and Erectile Function Enhancement

Various beverages, including red wine, green tea, pomegranate, blueberry, cranberry, and orange juice, are believed to be beneficial to erectile function in the context of ED by contributing to effective ROS scavenging [230,231]. In animal models of arteriogenic ED, pomegranate juice has demonstrated potential, reducing oxidative stress and enhancing erectile function, although it does not fully restore normal performance [232]. The beneficial effects of pomegranate may likely be attributed to its primary active components, most characteristically polyphenol antioxidants [233].

Resveratrol, a natural polyphenol predominantly present in red wine and grapes, has also demonstrated promise in animal models, restoring penile function in hypercholesterolemia- or diabetes-associated ED [234]. eNOS activation and improved endothelial function are the primary mechanisms to which the cardiovascular protective effects of resveratrol are attributed [71]. However, additional scientific and clinical research is required to comprehensively elucidate the specific mechanisms and effects of these commercially available antioxidants on penile oxidative stress and erectile performance.

## 6. Future Directions in Erectile Dysfunction Research

Future research into ED ought to investigate several key sites to enhance disease understanding and improve therapeutic approaches. One critical area involves the specific mechanisms that regulate the formation of ROS in the penis. By studying these mechanisms, researchers can gain valuable insights into the pathways responsible for oxidative stress-induced erectile dysfunction, potentially leading to the development of targeted therapies aimed at reducing oxidative damage and preserving erectile function [129].

Another promising direction for future research involves exploring the impact of environmental factors, such as PM2.5 exposure (Particulate Matter 2.5 μm or smaller), on oxidative stress and inflammatory responses in individuals with ED. Understanding how these environmental variables contribute to ED could uncover new therapeutic targets and strategies for managing the condition through environmental interventions [7].

The efficacy of antioxidants in reducing oxidative stress in ED is another area that requires further investigation. By assessing the impact of dietary antioxidants and plant extracts on arteriogenic ED, researchers could develop effective antioxidant-based treatment strategies. This approach may offer significant benefits to patients by mitigating oxidative stress and improving erectile function [231].

Furthermore, a deeper exploration of the relationship between nitric oxide signalling, oxidative stress, and endothelial dysfunction is crucial for a comprehensive understanding of ED. Future research should focus on the regulation of soluble guanylate cyclase and nitric oxide pathways, as this could lead to novel treatments that address the underlying mechanisms of ED [235].

Additionally, studying markers of oxidative stress and the structural integrity of nerves in chronic penile ischemia may provide insights into the neurodegenerative processes underlying ED. Understanding the effects of oxidative stress on penile neuropathy could reveal the underlying causes and lead to new treatment approaches that address these neurodegenerative aspects [174].

Finally, future research should examine the correlation between psychological factors, such as stress, depression, and cognitive interference, and oxidative stress in ED. By gaining a comprehensive understanding of how psychological factors influence oxidative stress levels, researchers can contribute to the adoption of a more holistic approach to managing ED, integrating both physiological and psychological interventions [236].

## 7. Conclusions

This study underscores the significant role of oxidative stress in the development and progression of ED, particularly through its impact on endothelial function and NO bioavailability. Key findings highlight that OS leads to endothelial dysfunction, smooth muscle cell apoptosis, and impaired NO production, which are crucial factors in the pathophysiology of ED. The analysis of various conditions such as aging, diabetes mellitus, hypertension, hyperlipidemia, chronic kidney disease, obesity, smoking, alcohol consumption, psychological stress, hyperhomocysteinemia, and sickle cell disease reveals a common pathway of increased ROS production contributing to endothelial dysfunction and reduced NO bioavailability.

Preclinical models demonstrate that antioxidants like glutathione, SOD, and catalase are crucial in mitigating oxidative damage. Therapeutic interventions, including high doses of vitamins E and C, hydrogen sulfide, and Angiotensin-(1–7), have shown promise in improving erectile function by countering oxidative stress and preventing fibrosis. However, the clinical application of antioxidant therapy faces challenges due to the non-specific action of antioxidants and their limited availability at target sites. Current evidence indicates that while antioxidant strategies have significant potential, their effectiveness in reversing ED is restricted. Further research is necessary to optimize antioxidant therapy, focusing on precise targeting of oxidative stress pathways without disrupting physiological functions. Refinement of antioxidant treatments to ensure their proper administration and delivery is crucial for developing robust therapeutic protocols.

## Figures and Tables

**Table 1:** Summary of ROS-generating sources in the context of ED conditions.

ROS-Generating Sources	Function and Mechanism	Conditions	Clinical and Experimental Models
NADPH oxidase	- Generates ROS by transferring electrons from NADPH to O₂ [54] - Comprises cytosolic (p47phox, p67phox) and membrane (p22phox, gp91phox) components [55,56]	Diabetes Mellitus, Hypertension, Cigarette Smoking, Sickle Cell Disease, Hyperhomocysteinemia, Obesity, Psychological Stress [57]	- Diabetes (rats) [114,117,118], - Hypertension (rats) [123,124], - Smoking (mice) [142,143], - Sickle Cell Disease (mice) [172], - Hyperhomocysteinemia (rabbits) [164,165], - Psychological Stress (rats) [161], - Obesity (human) [88]
eNOS uncoupling	- NOS isoforms (eNOS, nNOS, iNOS) typically produce NO - Under pathological conditions, produce superoxide (uncoupling) [67]	Aging, Diabetes Mellitus, Hyperlipidemia, Sickle Cell Disease, Cigarette Smoking, Chronic Kidney Disease [75]	- Aging (rats) [90], - Diabetes (mice) [112,113], - Hyperlipidemia (mice) [129,130], - SCD (mice) [172], - Obesity (human) [88], - Smoking (mice) [142,143], - Chronic Kidney Disease (mice) [135]
Mitochondrial ROS	- Produced during oxidative phosphorylation [79] - ETC in inner mitochondrial membrane transfers electrons through complexes I–IV [80]	Diabetes Mellitus, Hyperlipidemia [84]	- Diabetes Mellitus (mice) [84] - Hyperlipidemia (rats) [134]
Xanthine oxidase	- Converts hypoxanthine to xanthine, then uric acid - Generates ROS (hydrogen peroxide, superoxides) [60,61]	Diabetes Mellitus, Hyperlipidemia, Sickle Cell Disease [64]	- Diabetes (rats) [64], - Hyperlipidemia (rats) [134], - Sickle Cell Disease (mice) [172]

ROS: reactive oxygen species, NADPH: nicotinamide adenine dinucleotide phosphate, eNOS: endothelial nitric oxide synthase, iNOS: inducible nitric oxide synthase, nNOS: neuronal nitric oxide synthase, ETC: electron transport chain.

## Data Availability

No new data were created or analyzed in this study.

## References

[1] Elterman D.S., Bhattacharyya S.K., Mafilios M., Woodward E., Nitschelm K., Burnett A.L. (2021). The Quality of Life and Economic Burden of Erectile Dysfunction. Res. Rep. Urol..

[2] McMahon C.G. (2019). Current diagnosis and management of erectile dysfunction. Med. J. Aust..

[3] Irwin G.M. (2019). Erectile Dysfunction. Prim. Care.

[4] Sangiorgi G., Cereda A., Benedetto D., Bonanni M., Chiricolo G., Cota L., Martuscelli E., Greco F. (2021). Anatomy, Pathophysiology, Molecular Mechanisms, and Clinical Management of Erectile Dysfunction in Patients Affected by Coronary Artery Disease: A Review. Biomedicines.

[5] Ibrahim A., Ali M., Kiernan T.J., Stack A.G. (2018). Erectile Dysfunction and Ischaemic Heart Disease. Eur. Cardiol. Rev..

[6] Thomas C., Konstantinidis C. (2021). Neurogenic Erectile Dysfunction. Where Do We Stand?. Medicines.

[7] Roychoudhury S., Chakraborty S., Choudhury A.P., Das A., Jha N.K., Slama P., Nath M., Massanyi P., Ruokolainen J., Kesari K.K. (2021). Environmental Factors-Induced Oxidative Stress: Hormonal and Molecular Pathway Disruptions in Hypogonadism and Erectile Dysfunction. Antioxidants.

[8] Chen M., Zhang Z., Zhou R., Li B., Jiang J., Shi B. (2024). The relationship between oxidative balance score and erectile dysfunction in the U.S. male adult population. Sci. Rep..

[9] Chirindoth S.S., Cancarevic I. (2021). Role of Hydrogen Sulfide in the Treatment of Fibrosis. Cureus.

[10] Santos R.A.S., Sampaio W.O., Alzamora A.C., Motta-Santos D., Alenina N., Bader M., Campagnole-Santos M.J. (2018). The ACE2/Angiotensin-(1–7)/MAS Axis of the Renin-Angiotensin System: Focus on Angiotensin-(1–7). Physiol. Rev..

[11] MacDonald S.M., Burnett A.L. (2021). Physiology of Erection and Pathophysiology of Erectile Dysfunction. Urol. Clin. N. Am..

[12] Andersson K.E. (2019). Autonomic Regulation of Penile Erection.

[13] Mitidieri E., Cirino G., d’Emmanuele di Villa Bianca R., Sorrentino R. (2020). Pharmacology and perspectives in erectile dysfunction in man. Pharmacol. Ther..

[14] Melis M.R., Argiolas A. (2021). Erectile Function and Sexual Behavior: A Review of the Role of Nitric Oxide in the Central Nervous System. Biomolecules.

[15] Fujimoto K., Hashimoto D., Kashimada K., Kumegawa S., Ueda Y., Hyuga T., Hirashima T., Inoue N., Suzuki K., Hara I. (2022). A visualization system for erectile vascular dynamics. Front. Cell Dev. Biol..

[16] Hashimoto D., Hirashima T., Yamamura H., Kataoka T., Fujimoto K., Hyuga T., Yoshiki A., Kimura K., Kuroki S., Tachibana M. (2021). Dynamic erectile responses of a novel penile organ model utilizing TPEM^†^. Biol. Reprod..

[17] Burnett A.L. (2019). The Science and Practice of Erection Physiology: Story of a Revolutionary Gaseous Molecule. Trans. Am. Clin. Climatol. Assoc..

[18] Cripps S.M., Mattiske D.M., Pask A.J. (2021). Erectile Dysfunction in Men on the Rise: Is There a Link with Endocrine Disrupting Chemicals?. Sex. Dev..

[19] de Souza I.L.L., Ferreira E.D.S., Vasconcelos L.H.C., Cavalcante F.d.A., da Silva B.A. (2022). Erectile Dysfunction: Key Role of Cavernous Smooth Muscle Cells. Front. Pharmacol..

[20] Song S., Babicheva A., Zhao T., Ayon R.J., Rodriguez M., Rahimi S., Balistrieri F., Harrington A., Shyy J.Y.-J., Thistlethwaite P.A. (2020). Notch enhances Ca^2+^ entry by activating calcium-sensing receptors and inhibiting voltage-gated K^+^ channels. Am. J. Physiol. Cell Physiol..

[21] Ahmed W.S., Geethakumari A.M., Biswas K.H. (2021). Phosphodiesterase 5 (PDE5): Structure-function regulation and therapeutic applications of inhibitors. Biomed. Pharmacother..

[22] Pereira P.D.S., Pereira D.A., Calmasini F.B., Reis L.O., Brinkman N., Burnett A.L., Costa F.F., Silva F.H. (2022). Haptoglobin treatment contributes to regulating nitric oxide signal and reduces oxidative stress in the penis: A preventive treatment for priapism in sickle cell disease. Front. Physiol..

[23] Saenz de Tejada I., Angulo J., Cellek S., Gonzalez-Cadavid N., Heaton J., Pickard R., Simonsen U. (2004). Physiology of erectile function. J. Sex. Med..

[24] Andersson K.E. (2001). Neurophysiology/pharmacology of erection. Int. J. Impot. Res..

[25] Panchatsharam P.K., Durland J., Zito P.M. (2024). Physiology, Erection.

[26] Shindel A.W., Lue T.F., Feingold K.R., Anawalt B., Blackman M.R., Boyce A., Chrousos G., Corpas E., de Herder W.W., Dhatariya K., Dungan K., Hofland J. (2000). Medical and Surgical Therapy of Erectile Dysfunction.

[27] Aydinoglu F., Adibelli E.O., Yilmaz-Oral D., Ogulener N. (2019). Involvement of RhoA/Rho-kinase in l-cysteine/H_2_S pathway-induced inhibition of agonist-mediated corpus cavernosal smooth muscle contraction. Nitric Oxide.

[28] Wang H., Eto M., Steers W.D., Somlyo A.P., Somlyo A.V. (2002). RhoA-mediated Ca^2+^ sensitization in erectile function. J. Biol. Chem..

[29] Angulo J., Cuevas P., La Fuente J.M., Pomerol J.M., Ruiz-Castane E., Puigvert A., Gabancho S., Fernandez A., Ney P., Saenz De Tejada I. (2002). Regulation of human penile smooth muscle tone by prostanoid receptors. Br. J. Pharmacol..

[30] Gabani M., Liu J., Ait-Aissa K., Koval O., Kim Y.R., Castaneda D., Vikram A., Jacobs J.S., Grumbach I., Trebak M. (2019). MiR-204 regulates type 1 IP_3_R to control vascular smooth muscle cell contractility and blood pressure. Cell Calcium.

[31] Angulo J., Cuevas P., Fernandez A., Allona A., Moncada I., Martin-Morales A., La Fuente J.M., de Tejada I.S. (2006). Enhanced thromboxane receptor-mediated responses and impaired endothelium-dependent relaxation in human corpus cavernosum from diabetic impotent men: Role of protein kinase C activity. J. Pharmacol. Exp. Ther..

[32] McCabe M.P., Sharlip I.D., Atalla E., Balon R., Fisher A.D., Laumann E., Lee S.W., Lewis R., Segraves R.T. (2016). Definitions of Sexual Dysfunctions in Women and Men: A Consensus Statement From the Fourth International Consultation on Sexual Medicine 2015. J. Sex. Med..

[33] Kessler A., Sollie S., Challacombe B., Briggs K., Van Hemelrijck M. (2019). The global prevalence of erectile dysfunction: A review. BJU Int..

[34] Corona G., Lee D.M., Forti G., O’Connor D.B., Maggi M., O’Neill T.W., Pendleton N., Bartfai G., Boonen S., Casanueva F.F. (2010). Age-related changes in general and sexual health in middle-aged and older men: Results from the European Male Ageing Study (EMAS). J. Sex. Med..

[35] Ayta I.A., McKinlay J.B., Krane R.J. (1999). The likely worldwide increase in erectile dysfunction between 1995 and 2025 and some possible policy consequences. BJU Int..

[36] Molina-Vega M., Asenjo-Plaza M., Banderas-Donaire M.J., Hernandez-Ollero M.D., Rodriguez-Moreno S., Alvarez-Millan J.J., Cabezas-Sanchez P., Cardona-Diaz F., Alcaide-Torres J., Garrido-Sanchez L. (2020). Prevalence of and risk factors for erectile dysfunction in young nondiabetic obese men: Results from a regional study. Asian J. Androl..

[37] Cyr A.R., Huckaby L.V., Shiva S.S., Zuckerbraun B.S. (2020). Nitric Oxide and Endothelial Dysfunction. Crit. Care Clin..

[38] Krzastek S.C., Bopp J., Smith R.P., Kovac J.R. (2019). Recent advances in the understanding and management of erectile dysfunction. F1000Research.

[39] Scioli M.G., Storti G., D’Amico F., Rodriguez Guzman R., Centofanti F., Doldo E., Cespedes Miranda E.M., Orlandi A. (2020). Oxidative Stress and New Pathogenetic Mechanisms in Endothelial Dysfunction: Potential Diagnostic Biomarkers and Therapeutic Targets. J. Clin. Med..

[40] Das D., Shruthi N.R., Banerjee A., Jothimani G., Duttaroy A.K., Pathak S. (2023). Endothelial dysfunction, platelet hyperactivity, hypertension, and the metabolic syndrome: Molecular insights and combating strategies. Front. Nutr..

[41] Chung E. (2019). Contemporary and Novel Imaging Studies for the Evaluation of Erectile Dysfunction. Med. Sci..

[42] Zhang Y., Huo W., Wen Y., Li H. (2019). Silencing Nogo-B receptor inhibits penile corpus cavernosum vascular smooth muscle cell apoptosis of rats with diabetic erectile dysfunction by down-regulating ICAM-1. PLoS ONE.

[43] Doumas M., Tsakiris A., Douma S., Grigorakis A., Papadopoulos A., Hounta A., Tsiodras S., Dimitriou D., Giamarellou H. (2006). Factors affecting the increased prevalence of erectile dysfunction in Greek hypertensive compared with normotensive subjects. J. Androl..

[44] Schieber M., Chandel N.S. (2014). ROS function in redox signaling and oxidative stress. Curr. Biol..

[45] Thomas S.R., Witting P.K., Drummond G.R. (2008). Redox control of endothelial function and dysfunction: Molecular mechanisms and therapeutic opportunities. Antioxid. Redox Signal..

[46] Ma Z., Wang W., Pan C., Fan C., Li Y., Wang W., Lan T., Gong F., Zhao C., Zhao Z. (2022). N-acetylcysteine improves diabetic associated erectile dysfunction in streptozotocin-induced diabetic mice by inhibiting oxidative stress. J. Cell. Mol. Med..

[47] Taskiran M., Dogan K. (2023). The efficacy of systemic inflammatory response and oxidative stress in erectile dysfunction through multi-inflammatory index: A prospective cross-sectional analysis. J. Sex. Med..

[48] Mironczuk-Chodakowska I., Witkowska A.M., Zujko M.E. (2018). Endogenous non-enzymatic antioxidants in the human body. Adv. Med. Sci..

[49] Tabrez S., Ahmad M. (2011). Some enzymatic/nonenzymatic antioxidants as potential stress biomarkers of trichloroethylene, heavy metal mixture, and ethyl alcohol in rat tissues. Environ. Toxicol..

[50] Hamamcioglu A.C. (2017). The Role of Oxidative Stress and Antioxidants in Diabetes Mellitus. Turk. J. Diabetes Obes..

[51] Cervantes Gracia K., Llanas-Cornejo D., Husi H. (2017). CVD and Oxidative Stress. J. Clin. Med..

[52] Forstermann U., Xia N., Li H. (2017). Roles of Vascular Oxidative Stress and Nitric Oxide in the Pathogenesis of Atherosclerosis. Circ. Res..

[53] Holmstrom K.M., Finkel T. (2014). Cellular mechanisms and physiological consequences of redox-dependent signalling. Nat. Rev. Mol. Cell Biol..

[54] Duan J., Gao S., Tu S., Lenahan C., Shao A., Sheng J. (2021). Pathophysiology and Therapeutic Potential of NADPH Oxidases in Ischemic Stroke-Induced Oxidative Stress. Oxidative Med. Cell. Longev..

[55] Magnani F., Mattevi A. (2019). Structure and mechanisms of ROS generation by NADPH oxidases. Curr. Opin. Struct. Biol..

[56] Leto T.L., Morand S., Hurt D., Ueyama T. (2009). Targeting and regulation of reactive oxygen species generation by Nox family NADPH oxidases. Antioxid. Redox Signal..

[57] Schroder K. (2020). NADPH oxidases: Current aspects and tools. Redox Biol..

[58] Zhang Y., Murugesan P., Huang K., Cai H. (2020). NADPH oxidases and oxidase crosstalk in cardiovascular diseases: Novel therapeutic targets. Nat. Rev. Cardiol..

[59] Lee S.H., Lee M., Ko D.G., Choi B.Y., Suh S.W. (2021). The Role of NADPH Oxidase in Neuronal Death and Neurogenesis after Acute Neurological Disorders. Antioxidants.

[60] Bortolotti M., Polito L., Battelli M.G., Bolognesi A. (2021). Xanthine oxidoreductase: One enzyme for multiple physiological tasks. Redox Biol..

[61] Berry C.E., Hare J.M. (2004). Xanthine oxidoreductase and cardiovascular disease: Molecular mechanisms and pathophysiological implications. J. Physiol..

[62] Schmidt H.M., DeVallance E.R., Lewis S.E., Wood K.C., Annarapu G.K., Carreno M., Hahn S.A., Seman M., Maxwell B.A., Hileman E.A. (2023). Release of hepatic xanthine oxidase (XO) to the circulation is protective in intravascular hemolytic crisis. Redox Biol..

[63] Washio K., Kusunoki Y., Tsunoda T., Osugi K., Ohigashi M., Murase T., Nakamura T., Matsuo T., Konishi K., Katsuno T. (2020). Xanthine oxidoreductase activity correlates with vascular endothelial dysfunction in patients with type 1 diabetes. Acta Diabetol..

[64] Yang K.J., Choi W.J., Chang Y.K., Park C.W., Kim S.Y., Hong Y.A. (2023). Inhibition of Xanthine Oxidase Protects against Diabetic Kidney Disease through the Amelioration of Oxidative Stress via VEGF/VEGFR Axis and NOX-FoxO3a-eNOS Signaling Pathway. Int. J. Mol. Sci..

[65] Kim N.H., Hong B.K., Choi S.Y., Moo Kwon H., Cho C.S., Yi E.C., Kim W.U. (2013). Reactive oxygen species regulate context-dependent inhibition of NFAT5 target genes. Exp. Mol. Med..

[66] Kakimoto M., Fujii M., Sato I., Honma K., Nakayama H., Kirihara S., Fukuoka T., Ran S., Hirohata S., Kitamori K. (2023). Antioxidant action of xanthine oxidase inhibitor febuxostat protects the liver and blood vasculature in SHRSP5/Dmcr rats. J. Appl. Biomed..

[67] Katusic Z.S., d’Uscio L.V., Nath K.A. (2009). Vascular protection by tetrahydrobiopterin: Progress and therapeutic prospects. Trends Pharmacol. Sci..

[68] Karbach S., Wenzel P., Waisman A., Munzel T., Daiber A. (2014). eNOS uncoupling in cardiovascular diseases--the role of oxidative stress and inflammation. Curr. Pharm. Des..

[69] Janaszak-Jasiecka A., Siekierzycka A., Płoska A., Dobrucki I.T., Kalinowski L. (2021). Endothelial Dysfunction Driven by Hypoxia—The Influence of Oxygen Deficiency on NO Bioavailability. Biomolecules.

[70] Li H., Xia N., Hasselwander S., Daiber A. (2019). Resveratrol and Vascular Function. Int. J. Mol. Sci..

[71] Xia N., Forstermann U., Li H. (2014). Resveratrol and endothelial nitric oxide. Molecules.

[72] Yang Y.-M., Huang A., Kaley G., Sun D. (2009). eNOS uncoupling and endothelial dysfunction in aged vessels. Am. J. Physiol. Heart Circ. Physiol..

[73] De Pascali F., Hemann C., Samons K., Chen C.A., Zweier J.L. (2014). Hypoxia and reoxygenation induce endothelial nitric oxide synthase uncoupling in endothelial cells through tetrahydrobiopterin depletion and S-glutathionylation. Biochemistry.

[74] Galougahi K.K., Liu C.C., Gentile C., Kok C., Nunez A., Garcia A., Fry N.A.S., Davies M.J., Hawkins C.L., Rasmussen H.H. (2014). Glutathionylation Mediates Angiotensin II–Induced eNOS Uncoupling, Amplifying NADPH Oxidase-Dependent Endothelial Dysfunction. J. Am. Heart Assoc..

[75] Zhang Y., Janssens S.P., Wingler K., Schmidt H.H.H.W., Moens A.L. (2011). Modulating endothelial nitric oxide synthase: A new cardiovascular therapeutic strategy. Am. J. Physiol. Heart Circ. Physiol..

[76] Wang L., Zeng W., Wang C., Lu Y., Xiong X., Chen S., Huang Q., Yan F., Huang Q. (2024). SUMOylation and coupling of eNOS mediated by PIAS1 contribute to maintenance of vascular homeostasis. FASEB J..

[77] Singh U., Devaraj S., Vasquez-Vivar J., Jialal I. (2007). C-reactive protein decreases endothelial nitric oxide synthase activity via uncoupling. J. Mol. Cell. Cardiol..

[78] Shaito A., Aramouni K., Assaf R., Parenti A., Orekhov A., Yazbi A.E., Pintus G., Eid A.H. (2022). Oxidative Stress-Induced Endothelial Dysfunction in Cardiovascular Diseases. Front. Biosci..

[79] Stowe D.F., Camara A.K. (2009). Mitochondrial reactive oxygen species production in excitable cells: Modulators of mitochondrial and cell function. Antioxid. Redox Signal..

[80] Ortiz D., Forquer I., Boitz J., Soysa R., Elya C., Fulwiler A., Nilsen A., Polley T., Riscoe M.K., Ullman B. (2016). Targeting the Cytochrome bc1 Complex of Leishmania Parasites for Discovery of Novel Drugs. Antimicrob. Agents Chemother..

[81] Tseyang T., Valeros J., Vo P., Spinelli J.B. (2023). Oxygen-Independent Assays to Measure Mitochondrial Function in Mammals. J. Vis. Exp..

[82] Chua Y.L., Hagen T. (2011). Compound C prevents Hypoxia-Inducible Factor-1α protein stabilization by regulating the cellular oxygen availability via interaction with Mitochondrial Complex I. BMC Res. Notes.

[83] Chua Y.L., Dufour E., Dassa E.P., Rustin P., Jacobs H.T., Taylor C.T., Hagen T. (2010). Stabilization of hypoxia-inducible factor-1α protein in hypoxia occurs independently of mitochondrial reactive oxygen species production. J. Biol. Chem..

[84] Giacco F., Brownlee M. (2010). Oxidative stress and diabetic complications. Circ. Res..

[85] Hroudova J., Singh N., Fisar Z. (2014). Mitochondrial dysfunctions in neurodegenerative diseases: Relevance to Alzheimer’s disease. BioMed Res. Int..

[86] Chen Q., Camara A.K., Stowe D.F., Hoppel C.L., Lesnefsky E.J. (2007). Modulation of electron transport protects cardiac mitochondria and decreases myocardial injury during ischemia and reperfusion. Am. J. Physiol. Cell Physiol..

[87] Fujita N., Momota M., Ishida M., Iwane T., Hatakeyama S., Yoneyama T., Hashimoto Y., Yoshikawa K., Yamaya K., Ohyama C. (2022). Association of oxidative stress with erectile dysfunction in community-dwelling men and men on dialysis. Aging Male.

[88] Trebaticky B., Zitnanova I., Dvorakova M., Orszaghova Z., Paduchova Z., Durackova Z., Breza J., Muchova J. (2019). Role of oxidative stress, adiponectin and endoglin in the pathophysiology of erectile dysfunction in diabetic and non-diabetic men. Physiol. Res..

[89] Yu W., Wang J., Dai Y.T., Wang B., Xu Y., Gao Q.Q., Xu Z.P. (2022). Modulation of SIRT1 expression improves erectile function in aged rats. Asian J. Androl..

[90] Johnson J.M., Bivalacqua T.J., Lagoda G.A., Burnett A.L., Musicki B. (2011). eNOS-uncoupling in age-related erectile dysfunction. Int. J. Impot. Res..

[91] Ferrini M.G., Davila H.H., Valente E.G., Gonzalez-Cadavid N.F., Rajfer J. (2004). Aging-related induction of inducible nitric oxide synthase is vasculo-protective to the arterial media. Cardiovasc. Res..

[92] Bivalacqua T.J., Armstrong J.S., Biggerstaff J., Abdel-Mageed A.B., Kadowitz P.J., Hellstrom W.J., Champion H.C. (2003). Gene transfer of extracellular SOD to the penis reduces O2-* and improves erectile function in aged rats. Am. J. Physiol. Heart Circ. Physiol..

[93] Ferrini M., Magee T.R., Vernet D., Rajfer J., Gonzalez-Cadavid N.F. (2001). Aging-related expression of inducible nitric oxide synthase and markers of tissue damage in the rat penis. Biol. Reprod..

[94] Shi J.P., Zhao Y.M., Song Y.T. (2003). Effect of aging on expression of nitric oxide synthase I and activity of nitric oxide synthase in rat penis. Asian J. Androl..

[95] Gandhi J., Dagur G., Warren K., Smith N.L., Sheynkin Y.R., Zumbo A., Khan S.A. (2017). The Role of Diabetes Mellitus in Sexual and Reproductive Health: An Overview of Pathogenesis, Evaluation, and Management. Curr. Diabetes Rev..

[96] Burnett A.L., Strong T.D., Trock B.J., Jin L., Bivalacqua T.J., Musicki B. (2009). Serum biomarker measurements of endothelial function and oxidative stress after daily dosing of sildenafil in type 2 diabetic men with erectile dysfunction. J. Urol..

[97] Costa C., Soares R., Castela A., Adaes S., Hastert V., Vendeira P., Virag R. (2009). Increased endothelial apoptotic cell density in human diabetic erectile tissue—comparison with clinical data. J. Sex. Med..

[98] Esposito K., Ciotola M., Giugliano F., Sardelli L., Giugliano F., Maiorino M.I., Beneduce F., De Sio M., Giugliano D. (2008). Phenotypic assessment of endothelial microparticles in diabetic and nondiabetic men with erectile dysfunction. J. Sex. Med..

[99] Tuncayengin A., Biri H., Onaran M., Sen I., Tuncayengin O., Polat F., Erbas D., Bozkirli I. (2003). Cavernosal tissue nitrite, nitrate, malondialdehyde and glutathione levels in diabetic and non-diabetic erectile dysfunction. Int. J. Androl..

[100] Wan Z.H., Li W.Z., Li Y.Z., Chen L., Li G.H., Hu W.F., Peng S., Yu J.J., Guo F. (2011). Poly(ADP-Ribose) polymerase inhibition improves erectile function in diabetic rats. J. Sex. Med..

[101] Angulo J., Peiro C., Cuevas P., Gabancho S., Fernandez A., Gonzalez-Corrochano R., La Fuente J.M., Baron A.D., Chen K.S., de Tejada I.S. (2009). The novel antioxidant, AC3056 (2,6-di-t-butyl-4-((dimethyl-4-methoxyphenylsilyl)methyloxy)phenol), reverses erectile dysfunction in diabetic rats and improves NO-mediated responses in penile tissue from diabetic men. J. Sex. Med..

[102] Jin H.R., Kim W.J., Song J.S., Choi M.J., Piao S., Shin S.H., Tumurbaatar M., Tuvshintur B., Nam M.S., Ryu J.K. (2009). Functional and morphologic characterizations of the diabetic mouse corpus cavernosum: Comparison of a multiple low-dose and a single high-dose streptozotocin protocols. J. Sex. Med..

[103] Shukla N., Hotston M., Persad R., Angelini G.D., Jeremy J.Y. (2009). The administration of folic acid improves erectile function and reduces intracavernosal oxidative stress in the diabetic rabbit. BJU Int..

[104] Bivalacqua T.J., Usta M.F., Kendirci M., Pradhan L., Alvarez X., Champion H.C., Kadowitz P.J., Hellstrom W.J. (2005). Superoxide anion production in the rat penis impairs erectile function in diabetes: Influence of in vivo extracellular superoxide dismutase gene therapy. J. Sex. Med..

[105] De Young L., Yu D., Bateman R.M., Brock G.B. (2004). Oxidative stress and antioxidant therapy: Their impact in diabetes-associated erectile dysfunction. J. Androl..

[106] Paskaloglu K., Sener G., Ayangolu-Dulger G. (2004). Melatonin treatment protects against diabetes-induced functional and biochemical changes in rat aorta and corpus cavernosum. Eur. J. Pharmacol..

[107] Ryu J.K., Kim D.J., Lee T., Kang Y.S., Yoon S.M., Suh J.K. (2003). The role of free radical in the pathogenesis of impotence in streptozotocin-induced diabetic rats. Yonsei Med. J..

[108] Fatehi-Hassanabad Z., Chan C.B., Furman B.L. (2010). Reactive oxygen species and endothelial function in diabetes. Eur. J. Pharmacol..

[109] Picchi A., Capobianco S., Qiu T., Focardi M., Zou X., Cao J.M., Zhang C. (2010). Coronary microvascular dysfunction in diabetes mellitus: A review. World J. Cardiol..

[110] Musicki B., Kramer M.F., Becker R.E., Burnett A.L. (2005). Inactivation of phosphorylated endothelial nitric oxide synthase (Ser-1177) by O-GlcNAc in diabetes-associated erectile dysfunction. Proc. Natl. Acad. Sci. USA.

[111] Keegan A., Jack A.M., Cotter M.A., Cameron N.E. (2000). Effects of aldose reductase inhibition on responses of the corpus cavernosum and mesenteric vascular bed of diabetic rats. J. Cardiovasc. Pharmacol..

[112] Nangle M.R., Cotter M.A., Cameron N.E. (2010). Poly(ADP-ribose) polymerase inhibition reverses nitrergic neurovascular dysfunctions in penile erectile tissue from streptozotocin-diabetic mice. J. Sex. Med..

[113] Nangle M.R., Cotter M.A., Cameron N.E. (2006). IκB kinase 2 inhibition corrects defective nitrergic erectile mechanisms in diabetic mouse corpus cavernosum. Urology.

[114] Jin H.R., Kim W.J., Song J.S., Piao S., Choi M.J., Tumurbaatar M., Shin S.H., Yin G.N., Koh G.Y., Ryu J.K. (2011). Intracavernous delivery of a designed angiopoietin-1 variant rescues erectile function by enhancing endothelial regeneration in the streptozotocin-induced diabetic mouse. Diabetes.

[115] Chitaley K., Kupelian V., Subak L., Wessells H. (2009). Diabetes, obesity and erectile dysfunction: Field overview and research priorities. J. Urol..

[116] Kovanecz I., Ferrini M.G., Vernet D., Nolazco G., Rajfer J., Gonzalez-Cadavid N.F. (2006). Pioglitazone prevents corporal veno-occlusive dysfunction in a rat model of type 2 diabetes mellitus. BJU Int..

[117] Cellek S., Qu W., Schmidt A.M., Moncada S. (2004). Synergistic action of advanced glycation end products and endogenous nitric oxide leads to neuronal apoptosis in vitro: A new insight into selective nitrergic neuropathy in diabetes. Diabetologia.

[118] Cellek S., Foxwell N.A., Moncada S. (2003). Two phases of nitrergic neuropathy in streptozotocin-induced diabetic rats. Diabetes.

[119] Kloner R. (2007). Erectile dysfunction and hypertension. Int. J. Impot. Res..

[120] Touyz R.M. (2005). Intracellular mechanisms involved in vascular remodelling of resistance arteries in hypertension: Role of angiotensin II. Exp. Physiol..

[121] Ushiyama M., Morita T., Kuramochi T., Yagi S., Katayama S. (2004). Erectile dysfunction in hypertensive rats results from impairment of the relaxation evoked by neurogenic carbon monoxide and nitric oxide. Hypertens. Res..

[122] Ushiyama M., Kuramochi T., Yagi S., Katayama S. (2008). Antioxidant treatment with α-tocopherol improves erectile function in hypertensive rats. Hypertens. Res..

[123] Claudino M.A., Franco-Penteado C.F., Priviero F.B., Camargo E.A., Teixeira S.A., Muscara M.N., De Nucci G., Zanesco A., Antunes E. (2010). Upregulation of gp91phox subunit of NAD(P)H oxidase contributes to erectile dysfunction caused by long-term nitric oxide inhibition in rats: Reversion by regular physical training. Urology.

[124] Jin L., Lagoda G., Leite R., Webb R.C., Burnett A.L. (2008). NADPH oxidase activation: A mechanism of hypertension-associated erectile dysfunction. J. Sex. Med..

[125] Jeremy J.Y., Jones R.A., Koupparis A.J., Hotston M., Persad R., Angelini G.D., Shukla N. (2007). Reactive oxygen species and erectile dysfunction: Possible role of NADPH oxidase. Int. J. Impot. Res..

[126] Li R., Cui K., Wang T., Wang S., Li X., Qiu J., Yu G., Liu J., Wen B., Rao K. (2017). Hyperlipidemia impairs erectile function in rats by causing cavernosal fibrosis. Andrologia.

[127] Lee J.H., Oh J.H., Lee Y.J. (2012). Effects of experimental hyperlipidemia on the pharmacokinetics of tadalafil in rats. J. Pharm. Pharm. Sci..

[128] Huang Y.C., Ning H., Shindel A.W., Fandel T.M., Lin G., Harraz A.M., Lue T.F., Lin C.S. (2010). The effect of intracavernous injection of adipose tissue-derived stem cells on hyperlipidemia-associated erectile dysfunction in a rat model. J. Sex. Med..

[129] Musicki B., Liu T., Lagoda G.A., Strong T.D., Sezen S.F., Johnson J.M., Burnett A.L. (2010). Hypercholesterolemia-induced erectile dysfunction: Endothelial nitric oxide synthase (eNOS) uncoupling in the mouse penis by NAD(P)H oxidase. J. Sex. Med..

[130] Musicki B., Liu T., Strong T., Jin L., Laughlin M.H., Turk J.R., Burnett A.L. (2008). Low-fat diet and exercise preserve eNOS regulation and endothelial function in the penis of early atherosclerotic pigs: A molecular analysis. J. Sex. Med..

[131] Shukla N., Jones R., Persad R., Angelini G.D., Jeremy J.Y. (2005). Effect of sildenafil citrate and a nitric oxide donating sildenafil derivative, NCX 911, on cavernosal relaxation and superoxide formation in hypercholesterolaemic rabbits. Eur. J. Pharmacol..

[132] Zouaoui Boudjeltia K., Roumeguere T., Delree P., Moguilevsky N., Ducobu J., Vanhaeverbeek M., Wespes E. (2007). Presence of LDL modified by myeloperoxidase in the penis in patients with vascular erectile dysfunction: A preliminary study. Eur. Urol..

[133] Schulz E., Anter E., Keaney J.F. (2004). Oxidative stress, antioxidants, and endothelial function. Curr. Med. Chem..

[134] Zhong L., Ding W., Zeng Q., He B., Zhang H., Wang L., Fan J., He S., Zhang Y., Wei A. (2020). Sodium Tanshinone IIA Sulfonate Attenuates Erectile Dysfunction in Rats with Hyperlipidemia. Oxid. Med. Cell Longev..

[135] Podkowinska A., Formanowicz D. (2020). Chronic Kidney Disease as Oxidative Stress- and Inflammatory-Mediated Cardiovascular Disease. Antioxidants.

[136] Carlstrom M. (2021). Nitric oxide signalling in kidney regulation and cardiometabolic health. Nat. Rev. Nephrol..

[137] Duni A., Liakopoulos V., Roumeliotis S., Peschos D., Dounousi E. (2019). Oxidative Stress in the Pathogenesis and Evolution of Chronic Kidney Disease: Untangling Ariadne’s Thread. Int. J. Mol. Sci..

[138] Roumeliotis S., Mallamaci F., Zoccali C. (2020). Endothelial Dysfunction in Chronic Kidney Disease, from Biology to Clinical Outcomes: A 2020 Update. J. Clin. Med..

[139] Fontecha-Barriuso M., Lopez-Diaz A.M., Guerrero-Mauvecin J., Miguel V., Ramos A.M., Sanchez-Nino M.D., Ruiz-Ortega M., Ortiz A., Sanz A.B. (2022). Tubular Mitochondrial Dysfunction, Oxidative Stress, and Progression of Chronic Kidney Disease. Antioxidants.

[140] Jabarpour M., Rashtchizadeh N., Argani H., Ghorbanihaghjo A., Ranjbarzadhag M., Sanajou D., Panah F., Alirezaei A. (2019). The impact of dyslipidemia and oxidative stress on vasoactive mediators in patients with renal dysfunction. Int. Urol. Nephrol..

[141] Allen M.S., Tostes R.C. (2023). Cigarette smoking and erectile dysfunction: An updated review with a focus on pathophysiology, e-cigarettes, and smoking cessation. Sex. Med. Rev..

[142] Dikalov S., Itani H., Richmond B., Vergeade A., Rahman S.M.J., Boutaud O., Blackwell T., Massion P.P., Harrison D.G., Dikalova A. (2019). Tobacco smoking induces cardiovascular mitochondrial oxidative stress, promotes endothelial dysfunction, and enhances hypertension. Am. J. Physiol. Heart Circ. Physiol..

[143] Barbieri S.S., Zacchi E., Amadio P., Gianellini S., Mussoni L., Weksler B.B., Tremoli E. (2011). Cytokines present in smokers’ serum interact with smoke components to enhance endothelial dysfunction. Cardiovasc. Res..

[144] Imamura M., Waseda Y., Marinova G.V., Ishibashi T., Obayashi S., Sasaki A., Nagai A., Azuma H. (2007). Alterations of NOS, arginase, and DDAH protein expression in rabbit cavernous tissue after administration of cigarette smoke extract. Am. J. Physiol. Regul. Integr. Comp. Physiol..

[145] Lin J.H., Ho D.R., Shi C.S., Chen C.S., Li J.M., Huang Y.C. (2020). The influence of smoking exposure and cessation on penile hemodynamics and corporal tissue in a rat model. Transl. Androl. Urol..

[146] Hotston M.R., Jeremy J.Y., Bloor J., Koupparis A., Persad R., Shukla N. (2007). Sildenafil inhibits the up-regulation of phosphodiesterase type 5 elicited with nicotine and tumour necrosis factor-α in cavernosal vascular smooth muscle cells: Mediation by superoxide. BJU Int..

[147] Virdis A., Masi S., Colucci R., Chiriaco M., Uliana M., Puxeddu I., Bernardini N., Blandizzi C., Taddei S. (2019). Microvascular Endothelial Dysfunction in Patients with Obesity. Curr. Hypertens. Rep..

[148] Marseglia L., Manti S., D’Angelo G., Nicotera A., Parisi E., Di Rosa G., Gitto E., Arrigo T. (2014). Oxidative stress in obesity: A critical component in human diseases. Int. J. Mol. Sci..

[149] Kajikawa M., Higashi Y. (2022). Obesity and Endothelial Function. Biomedicines.

[150] Paduch D.A., Bolyakov A., Vaucher L. (2020). Obesity and sexual dysfunction in men. Obesity and Gynecology.

[151] Soardo G., Donnini D., Varutti R., Moretti M., Milocco C., Basan L., Esposito W., Casaccio D., Stel G., Catena C. (2005). Alcohol-induced endothelial changes are associated with oxidative stress and are rapidly reversed after withdrawal. Alcohol. Clin. Exp. Res..

[152] Phillips S.A., Osborn K., Hwang C.L., Sabbahi A., Piano M.R. (2020). Ethanol Induced Oxidative Stress in the Vasculature: Friend or Foe. Curr. Hypertens. Rev..

[153] Tan H.K., Yates E., Lilly K., Dhanda A.D. (2020). Oxidative stress in alcohol-related liver disease. World J. Hepatol..

[154] Finelli R., Mottola F., Agarwal A. (2021). Impact of Alcohol Consumption on Male Fertility Potential: A Narrative Review. Int. J. Environ. Res. Public Health.

[155] Kamal H., Tan G.C., Ibrahim S.F., Shaikh M.F., Mohamed I.N., Mohamed R.M.P., Hamid A.A., Ugusman A., Kumar J. (2020). Alcohol Use Disorder, Neurodegeneration, Alzheimer’s and Parkinson’s Disease: Interplay Between Oxidative Stress, Neuroimmune Response and Excitotoxicity. Front. Cell Neurosci..

[156] Li S., Song J.M., Zhang K., Zhang C.L. (2021). A Meta-Analysis of Erectile Dysfunction and Alcohol Consumption. Urol. Int..

[157] Karunakaran A., Prabhakaran A., Karunakaran V., Michael J.P. (2024). Erectile Dysfunction in Alcohol Use Disorder and the change in erectile function after one month of abstinence. J. Addict. Dis..

[158] Tsigos C., Kyrou I., Kassi E., Chrousos G.P., Feingold K.R., Anawalt B., Blackman M.R., Boyce A., Chrousos G., Corpas E., de Herder W.W., Dhatariya K., Dungan K., Hofland J. (2000). Stress: Endocrine Physiology and Pathophysiology.

[159] Chu B., Marwaha K., Sanvictores T., Awosika A.O., Ayers D. (2024). Physiology, Stress Reaction.

[160] Salim S. (2014). Oxidative stress and psychological disorders. Curr. Neuropharmacol..

[161] Schiavone S., Sorce S., Dubois-Dauphin M., Jaquet V., Colaianna M., Zotti M., Cuomo V., Trabace L., Krause K.H. (2009). Involvement of NOX2 in the development of behavioral and pathologic alterations in isolated rats. Biol. Psychiatry.

[162] Xiao Y., Xie T., Peng J., Zhou X., Long J., Yang M., Zhu H., Yang J. (2023). Factors associated with anxiety and depression in patients with erectile dysfunction: A cross-sectional study. BMC Psychol..

[163] Salvio G., Ciarloni A., Cutini M., Balercia G. (2021). Hyperhomocysteinemia: Focus on Endothelial Damage as a Cause of Erectile Dysfunction. Int. J. Mol. Sci..

[164] Koupparis A.J., Jeremy J., Angelini G., Persad R., Shukla N. (2006). Penicillamine administration reverses the inhibitory effect of hyperhomocysteinaemia on endothelium-dependent relaxation in the corpus cavernosum in the rabbit. BJU Int..

[165] Jones R.W., Jeremy J.Y., Koupparis A., Persad R., Shukla N. (2005). Cavernosal dysfunction in a rabbit model of hyperhomocysteinaemia. BJU Int..

[166] Kato G.J., Hebbel R.P., Steinberg M.H., Gladwin M.T. (2009). Vasculopathy in sickle cell disease: Biology, pathophysiology, genetics, translational medicine, and new research directions. Am. J. Hematol..

[167] Nader E., Romana M., Connes P. (2020). The Red Blood Cell—Inflammation Vicious Circle in Sickle Cell Disease. Front. Immunol..

[168] Chirico E.N., Pialoux V. (2012). Role of oxidative stress in the pathogenesis of sickle cell disease. IUBMB Life.

[169] Wood K.C., Hsu L.L., Gladwin M.T. (2008). Sickle cell disease vasculopathy: A state of nitric oxide resistance. Free Radic. Biol. Med..

[170] Idris I.M., Burnett A.L., DeBaun M.R. (2022). Epidemiology and treatment of priapism in sickle cell disease. Hematol. Am. Soc. Hematol. Educ. Program.

[171] Chinegwundoh F.I., Smith S., Anie K.A. (2020). Treatments for priapism in boys and men with sickle cell disease. Cochrane Database Syst. Rev..

[172] Kanika N.D., Melman A., Davies K.P. (2010). Experimental priapism is associated with increased oxidative stress and activation of protein degradation pathways in corporal tissue. Int. J. Impot. Res..

[173] Agarwal A., Nandipati K.C., Sharma R.K., Zippe C.D., Raina R. (2006). Role of oxidative stress in the pathophysiological mechanism of erectile dysfunction. J. Androl..

[174] Azadzoi K.M., Golabek T., Radisavljevic Z.M., Yalla S.V., Siroky M.B. (2010). Oxidative stress and neurodegeneration in penile ischaemia. BJU Int..

[175] Uluocak N., Atilgan D., Erdemir F., Parlaktas B.S., Yasar A., Erkorkmaz U., Akbas A. (2010). An animal model of ischemic priapism and the effects of melatonin on antioxidant enzymes and oxidative injury parameters in rat penis. Int. Urol. Nephrol..

[176] Lagoda G., Jin L., Lehrfeld T.J., Liu T., Burnett A.L. (2007). FK506 and sildenafil promote erectile function recovery after cavernous nerve injury through antioxidative mechanisms. J. Sex. Med..

[177] Yeni E., Gulum M., Selek S., Erel O., Unal D., Verit A., Savas M. (2005). Comparison of oxidative/antioxidative status of penile corpus cavernosum blood and peripheral venous blood. Int. J. Impot. Res..

[178] Kawakami T., Urakami S., Hirata H., Tanaka Y., Nakajima K., Enokida H., Shiina H., Ogishima T., Tokizane T., Kawamoto K. (2009). Superoxide dismutase analog (Tempol: 4-hydroxy-2, 2, 6, 6-tetramethylpiperidine 1-oxyl) treatment restores erectile function in diabetes-induced impotence. Int. J. Impot. Res..

[179] Kim S.C., Kim I.K., Seo K.K., Baek K.J., Lee M.Y. (1997). Involvement of superoxide radical in the impaired endothelium-dependent relaxation of cavernous smooth muscle in hypercholesterolemic rabbits. Urol. Res..

[180] Kaltsas A. (2023). Oxidative Stress and Male Infertility: The Protective Role of Antioxidants. Medicina.

[181] Zhou B., Chen Y., Yuan H., Wang T., Feng J., Li M., Liu J. (2021). NOX1/4 Inhibitor GKT-137831 Improves Erectile Function in Diabetic Rats by ROS Reduction and Endothelial Nitric Oxide Synthase Reconstitution. J. Sex. Med..

[182] Tostes R.C., Carneiro F.S., Lee A.J., Giachini F.R., Leite R., Osawa Y., Webb R.C. (2008). Cigarette smoking and erectile dysfunction: Focus on NO bioavailability and ROS generation. J. Sex. Med..

[183] Paulis G., De Giorgio G. (2022). Full Regression of Peyronie’s Disease Plaque Following Combined Antioxidant Treatment: A Three-Case Report. Antioxidants.

[184] Fraga-Silva R.A., Costa-Fraga F.P., Savergnini S.Q., De Sousa F.B., Montecucco F., da Silva D., Sinisterra R.D., Mach F., Stergiopulos N., da Silva R.F. (2013). An oral formulation of angiotensin-(1-7) reverses corpus cavernosum damages induced by hypercholesterolemia. J. Sex. Med..

[185] La Favor J.D., Rhein P.J., Pierre C.J., Azeez T., Burnett A.L. (2023). (090) Hydrogen Sulfide Therapy Stimulates Cellular Antioxidant Defense and Reverses Erectile Dysfunction in Western Diet-fed Mice. J. Sex. Med..

[186] Sheweita S.A., Meftah A.A., Sheweita M.S., Balbaa M.E. (2020). Erectile dysfunction drugs altered the activities of antioxidant enzymes, oxidative stress and the protein expressions of some cytochrome P450 isozymes involved in the steroidogenesis of steroid hormones. PLoS ONE.

[187] Fu H., Bai X., Le L., Tian D., Gao H., Qi L.X., Hu K.P. (2019). Eucommia ulmoides Oliv. Leaf Extract Improves Erectile Dysfunction in Streptozotocin-Induced Diabetic Rats by Protecting Endothelial Function and Ameliorating Hypothalamic-Pituitary-Gonadal Axis Function. Evid. Based Complement. Alternat Med..

[188] Jeffrey S., Samraj P.I., Raj B.S. (2021). The Role of Alpha-lipoic Acid Supplementation in the Prevention of Diabetes Complications: A Comprehensive Review of Clinical Trials. Curr. Diabetes Rev..

[189] Tadayon Najafabadi B., Jafarinia M., Ghamari K., Shokraee K., Tadayyon F., Akhondzadeh S. (2019). Vitamin E and ginseng combined supplement for treatment of male erectile dysfunction: A double-blind, placebo-controlled, randomized, clinical trial. Adv. Integr. Med..

[190] Tang Z., Song J., Yu Z., Cui K., Ruan Y., Wang T., Yang J., Wang S., Liu J. (2019). Melatonin Treatment Ameliorates Hyperhomocysteinemia-Induced Impairment of Erectile Function in a Rat Model. J. Sex. Med..

[191] Shivavedi N., Charan Tej G.N.V., Neogi K., Nayak P.K. (2019). Ascorbic acid therapy: A potential strategy against comorbid depression-like behavior in streptozotocin-nicotinamide-induced diabetic rats. Biomed. Pharmacother..

[192] Wang W., Kang P.M. (2020). Oxidative Stress and Antioxidant Treatments in Cardiovascular Diseases. Antioxidants.

[193] Song J., Tang Z., Li H., Jiang H., Sun T., Lan R., Wang T., Wang S., Ye Z., Liu J. (2019). Role of JAK2 in the Pathogenesis of Diabetic Erectile Dysfunction and an Intervention With Berberine. J. Sex. Med..

[194] Madeira C.R., Tonin F.S., Fachi M.M., Borba H.H., Ferreira V.L., Leonart L.P., Bonetti A.F., Moritz R.P., Trindade A.C.L.B., Goncalves A.G. (2021). Efficacy and safety of oral phosphodiesterase 5 inhibitors for erectile dysfunction: A network meta-analysis and multicriteria decision analysis. World J. Urol..

[195] Sofikitis N., Kaltsas A., Dimitriadis F., Rassweiler J., Grivas N., Zachariou A., Kaponis A., Tsounapi P., Paterakis N., Karagiannis A. (2021). The Effect of PDE5 Inhibitors on the Male Reproductive Tract. Curr. Pharm. Des..

[196] Dimitriadis F., Kaltsas A., Zachariou A., Mamoulakis C., Tsiampali C., Giannakis I., Paschopoulos M., Papatsoris A., Loutradis D., Tsounapi P. (2022). PDE5 inhibitors and male reproduction: Is there a place for PDE5 inhibitors in infertility clinics or andrology laboratories?. Int. J. Urol..

[197] Pyrgidis N., Mykoniatis I., Haidich A.B., Tirta M., Talimtzi P., Kalyvianakis D., Ouranidis A., Hatzichristou D. (2021). Effect of phosphodiesterase-type 5 inhibitors on erectile function: An overview of systematic reviews and meta-analyses. BMJ Open.

[198] Bivalacqua T.J., Sussan T.E., Gebska M.A., Strong T.D., Berkowitz D.E., Biswal S., Burnett A.L., Champion H.C. (2009). Sildenafil inhibits superoxide formation and prevents endothelial dysfunction in a mouse model of secondhand smoke induced erectile dysfunction. J. Urol..

[199] Hotston M., Jeremy J.Y., Bloor J., Greaves N.S., Persad R., Angelini G., Shukla N. (2008). Homocysteine and copper interact to promote type 5 phosphodiesterase expression in rabbit cavernosal smooth muscle cells. Asian J. Androl..

[200] Koupparis A.J., Jeremy J.Y., Muzaffar S., Persad R., Shukla N. (2005). Sildenafil inhibits the formation of superoxide and the expression of gp47^phox^ NAD[P]H oxidase induced by the thromboxane A2 mimetic, U46619, in corpus cavernosal smooth muscle cells. BJU Int..

[201] Tzoumas N., Farrah T.E., Dhaun N., Webb D.J. (2020). Established and emerging therapeutic uses of PDE type 5 inhibitors in cardiovascular disease. Br. J. Pharmacol..

[202] Shukla N., Rossoni G., Hotston M., Sparatore A., Del Soldato P., Tazzari V., Persad R., Angelini G.D., Jeremy J.Y. (2009). Effect of hydrogen sulphide-donating sildenafil (ACS6) on erectile function and oxidative stress in rabbit isolated corpus cavernosum and in hypertensive rats. BJU Int..

[203] Saikia Q., Hazarika A.K., Mishra R. (2022). A Review on the Pharmacological Importance of PDE5 and Its Inhibition to Manage Biomedical Conditions. J. Pharmacol. Pharmacother..

[204] Barbagallo F., Campolo F., Franceschini E., Crecca E., Pofi R., Isidori A.M., Venneri M.A. (2020). PDE5 Inhibitors in Type 2 Diabetes Cardiovascular Complications. Endocrines.

[205] Verit A., Savas M., Ciftci H., Aksoy N., Taskin A., Topal U. (2010). Assessment of the acute effects of tadalafil on the cardiovascular system based on examination of serum oxidative status and paraoxonase activity in men with erectile dysfunction: A preliminary study. Int. J. Impot. Res..

[206] Lombardo R., Tema G., De Nunzio C. (2021). Phosphodiesterases 5 Inhibitors and Erectile Dysfunction Recovery after Pelvic Surgery: Future Perspectives for New Drugs and New Formulations. Curr. Drug Targets.

[207] Deger M.D., Madendere S. (2021). Erectile dysfunction treatment with Phosphodiesterase-5 Inhibitors: Google trends analysis of last 10 years and COVID-19 pandemic. Arch. Ital. Urol. Androl..

[208] Ding J., Yu M., Jiang J., Luo Y., Zhang Q., Wang S., Yang F., Wang A., Wang L., Zhuang M. (2020). Angiotensin II Decreases Endothelial Nitric Oxide Synthase Phosphorylation via AT(1)R Nox/ROS/PP2A Pathway. Front. Physiol..

[209] Friedrich E.B., Teo K.K., Bohm M. (2006). ACE inhibition in secondary prevention: Are the results controversial?. Clin. Res. Cardiol..

[210] do Vale G.T., Tirapelli C.R. (2020). Are Reactive Oxygen Species Important Mediators of Vascular Dysfunction?. Curr. Hypertens. Rev..

[211] Nunes K.P., Labazi H., Webb R.C. (2012). New insights into hypertension-associated erectile dysfunction. Curr. Opin. Nephrol. Hypertens..

[212] Baumhakel M., Custodis F., Schlimmer N., Laufs U., Bohm M. (2008). Improvement of endothelial function of the corpus cavernosum in apolipoprotein E knockout mice treated with irbesartan. J. Pharmacol. Exp. Ther..

[213] Park K., Shin J.W., Oh J.K., Ryu K.S., Kim S.W., Paick J.S. (2005). Restoration of erectile capacity in normotensive aged rats by modulation of angiotensin receptor type 1. J. Androl..

[214] Idris Khodja N., Chataigneau T., Auger C., Schini-Kerth V.B. (2012). Grape-derived polyphenols improve aging-related endothelial dysfunction in rat mesenteric artery: Role of oxidative stress and the angiotensin system. PLoS ONE.

[215] Chen F.P., Gong L.K., Zhang L., Wang H., Qi X.M., Wu X.F., Xiao Y., Cai Y., Liu L.L., Li X.H. (2007). Early lung injury contributes to lung fibrosis via AT1 receptor in rats. Acta Pharmacol. Sin..

[216] Yanagitani Y., Rakugi H., Okamura A., Moriguchi K., Takiuchi S., Ohishi M., Suzuki K., Higaki J., Ogihara T. (1999). Angiotensin II type 1 receptor-mediated peroxide production in human macrophages. Hypertension.

[217] Zinellu A., Mangoni A.A. (2021). A Systematic Review and Meta-Analysis of the Effect of Statins on Glutathione Peroxidase, Superoxide Dismutase, and Catalase. Antioxidants.

[218] Adam O., Laufs U. (2008). Antioxidative effects of statins. Arch. Toxicol..

[219] Wassmann S., Laufs U., Baumer A.T., Muller K., Ahlbory K., Linz W., Itter G., Rosen R., Bohm M., Nickenig G. (2001). HMG-CoA reductase inhibitors improve endothelial dysfunction in normocholesterolemic hypertension via reduced production of reactive oxygen species. Hypertension.

[220] Wenzel P., Daiber A., Oelze M., Brandt M., Closs E., Xu J., Thum T., Bauersachs J., Ertl G., Zou M.H. (2008). Mechanisms underlying recoupling of eNOS by HMG-CoA reductase inhibition in a rat model of streptozotocin-induced diabetes mellitus. Atherosclerosis.

[221] Park B.H., Han D.-S., Yuk S.M., Youn C.S., Kwon E.B., Park K.C., Jang H. (2020). Preservation of erectile function by statins in a rat model of erectile dysfunction induced by hypercholesterolemia. J. Men’s Health.

[222] Miner M., Billups K.L. (2008). Erectile dysfunction and dyslipidemia: Relevance and role of phosphodiesterase type-5 inhibitors and statins. J. Sex. Med..

[223] Hong S.K., Han B.K., Jeong S.J., Byun S.S., Lee S.E. (2007). Effect of statin therapy on early return of potency after nerve sparing radical retropubic prostatectomy. J. Urol..

[224] La Vignera S., Condorelli R.A., Vicari E., Calogero A.E. (2012). Statins and erectile dysfunction: A critical summary of current evidence. J. Androl..

[225] Rizvi K., Hampson J.P., Harvey J.N. (2002). Do lipid-lowering drugs cause erectile dysfunction? A systematic review. Fam. Pract..

[226] Solomon H., Samarasinghe Y.P., Feher M.D., Man J., Rivas-Toro H., Lumb P.J., Wierzbicki A.S., Jackson G. (2006). Erectile dysfunction and statin treatment in high cardiovascular risk patients. Int. J. Clin. Pract..

[227] Bendall J.K., Douglas G., McNeill E., Channon K.M., Crabtree M.J. (2014). Tetrahydrobiopterin in cardiovascular health and disease. Antioxid. Redox Signal..

[228] Chuaiphichai S., Chu S.M., Carnicer R., Kelly M., Bendall J.K., Simon J.N., Douglas G., Crabtree M.J., Casadei B., Channon K.M. (2023). Endothelial cell-specific roles for tetrahydrobiopterin in myocardial function, cardiac hypertrophy, and response to myocardial ischemia-reperfusion injury. Am. J. Physiol. Heart Circ. Physiol..

[229] Chen D.D., Chen L.Y., Xie J.B., Shu C., Yang T., Zhou S., Yuan H., Chen A.F. (2014). Tetrahydrobiopterin regulation of eNOS redox function. Curr. Pharm. Des..

[230] Rudrapal M., Khairnar S.J., Khan J., Dukhyil A.B., Ansari M.A., Alomary M.N., Alshabrmi F.M., Palai S., Deb P.K., Devi R. (2022). Dietary Polyphenols and Their Role in Oxidative Stress-Induced Human Diseases: Insights Into Protective Effects, Antioxidant Potentials and Mechanism(s) of Action. Front. Pharmacol..

[231] Zhang Q., Radisavljevic Z.M., Siroky M.B., Azadzoi K.M. (2011). Dietary antioxidants improve arteriogenic erectile dysfunction. Int. J. Androl..

[232] Azadzoi K.M., Schulman R.N., Aviram M., Siroky M.B. (2005). Oxidative stress in arteriogenic erectile dysfunction: Prophylactic role of antioxidants. J. Urol..

[233] Zarfeshany A., Asgary S., Javanmard S.H. (2014). Potent health effects of pomegranate. Adv. Biomed. Res..

[234] Weiskirchen S., Weiskirchen R. (2016). Resveratrol: How Much Wine Do You Have to Drink to Stay Healthy?. Adv. Nutr..

[235] Lasker G.F., Pankey E.A., Kadowitz P.J. (2013). Modulation of soluble guanylate cyclase for the treatment of erectile dysfunction. Physiology.

[236] Allen M.S., Wood A.M., Sheffield D. (2023). The Psychology of Erectile Dysfunction. Curr. Dir. Psychol. Sci..

